# Mechanisms Underlying the Strong Inhibition of Muscle-Type Nicotinic Receptors by Tetracaine

**DOI:** 10.3389/fnmol.2018.00193

**Published:** 2018-08-08

**Authors:** Raúl Cobo, Magdalena Nikolaeva, Armando Alberola-Die, Gregorio Fernández-Ballester, José M. González-Ros, Isabel Ivorra, Andrés Morales

**Affiliations:** ^1^División de Fisiología, Departamento de Fisiología, Genética y Microbiología, Universidad de Alicante, Alicante, Spain; ^2^Instituto de Biología Molecular y Celular, Universidad Miguel Hernández, Alicante, Spain

**Keywords:** tetracaine, nicotinic acetylcholine receptors, *Xenopus* oocytes, microtransplanted receptors, desensitization, mechanisms of blockade

## Abstract

Nicotinic acetylcholine (ACh) receptors (nAChRs) are included among the targets of a variety of local anesthetics, although the molecular mechanisms of blockade are still poorly understood. Some local anesthetics, such as lidocaine, act on nAChRs by different means through their ability to present as both charged and uncharged molecules. Thus, we explored the mechanisms of nAChR blockade by tetracaine, which at physiological pH is almost exclusively present as a positively charged local anesthetic. The nAChRs from *Torpedo* electroplaques were transplanted to *Xenopus* oocytes and the currents elicited by ACh (*I*_*ACh*_s), either alone or co-applied with tetracaine, were recorded. Tetracaine reversibly blocked *I*_*ACh*_, with an *IC*_*50*_ (i.e., the concentration required to inhibit half the maximum *I*_*ACh*_) in the submicromolar range. Notably, at very low concentrations (0.1 μM), tetracaine reduced *I*_*ACh*_ in a voltage-dependent manner, the more negative potentials produced greater inhibition, indicating open-channel blockade. When the tetracaine concentration was increased to 0.7 μM or above, voltage-independent inhibition was also observed, indicating closed-channel blockade. The *I*_*ACh*_ inhibition by pre-application of just 0.7 μM tetracaine before superfusion of ACh also corroborated the notion of tetracaine blockade of resting nAChRs. Furthermore, tetracaine markedly increased nAChR desensitization, mainly at concentrations equal or higher than 0.5 μM. Interestingly, tetracaine did not modify desensitization when its binding within the channel pore was prevented by holding the membrane at positive potentials. Tetracaine-nAChR interactions were assessed by virtual docking assays, using nAChR models in the closed and open states. These assays revealed that tetracaine binds at different sites of the nAChR located at the extracellular and transmembrane domains, in both open and closed conformations. Extracellular binding sites seem to be associated with closed-channel blockade; whereas two sites within the pore, with different affinities for tetracaine, contribute to open-channel blockade and the enhancement of desensitization, respectively. These results demonstrate a concentration-dependent heterogeneity of tetracaine actions on nAChRs, and contribute to a better understanding of the complex modulation of muscle-type nAChRs by local anesthetics. Furthermore, the combination of functional and virtual assays to decipher nAChR-tetracaine interactions has allowed us to tentatively assign the main nAChR residues involved in these modulating actions.

## Introduction

The muscle-type nicotinic acetylcholine (ACh) receptor (nAChR) is the prototypical member of the Cys-loop family of ligand-gated ion channels. This receptor is a heteropentameric protein that is highly expressed by muscle fibers at the neuromuscular junction, and it is composed of 2α_1_, β_1_, δ, and ε (substituted by γ during fetal life or in denervated fibers) subunits that are arranged to form a central channel pore (Albuquerque et al., 2009). From a functional point of view, nAChRs are key elements for striated-muscle activation by motoneurons, and thus, for executing voluntary movements. The nAChRs are also expressed in both the central and peripheral nervous systems, and even in non-neuronal tissues, such as astrocytes, keratinocytes, lymphoid cells, lung epithelial cells, and vascular smooth muscle and endothelial cells (Gotti and Clementi, 2004). Although all nAChRs share many structural properties, neuronal nAChRs differ from their muscle-type counterparts in the large diversity of their subunit compositions, which in some cases are tissue specific, and in the associated heterogeneity of their physiological and pharmacological properties (Albuquerque et al., 2009; Taly et al., 2009). Remarkably, these receptors constitute a key therapeutic target, given the high prevalence and relevance of disorders related to nAChR dysfunction, including some myasthenias, addictive behaviors, some types of epilepsy, schizophrenia, Parkinson's and Alzheimer's diseases, inflammation, pain, and even cancer (Hurst et al., 2013; Wu et al., 2015; Parikh et al., 2016; Schulte et al., 2016). Therefore, over the last few decades, much effort has been devoted to understand the mechanisms underlying nAChR modulation, as a large number of highly different molecules affect their functional properties, enabling these receptors to act as allosteric proteins (Changeux, 2014).

Local anesthetics (LAs) are listed among the molecules known to inhibit nAChR activity, including some that are widely used in clinical practice, such as lidocaine (Steinbach, 1968; Wang et al., 2010; Alberola-Die et al., 2011), procaine (Katz and Miledi, 1975; Adams, 1977; Gage and Wachtel, 1984), tetracaine (Ttc) (Koblin and Lester, 1979; Gallagher and Cohen, 1999; Gentry and Lukas, 2001), bupivacaine (Ikeda et al., 1984), benzocaine (Koblin and Lester, 1979; Ogden et al., 1981), adiphenine, and proadifen (Gentry and Lukas, 2001; Spitzmaul et al., 2009). Most LAs seem to be able to inhibit nAChRs; however, there are marked differences among their molecular structures and potencies for nAChR blockade, suggesting that they might not bind to the same modulating sites on these receptors, which would explain their heterogeneous actions on nAChRs. Notably, we have found that lidocaine exerts multiple inhibitory actions on muscle- and neuronal-type nAChRs (Alberola-Die et al., 2011, 2013). Furthermore, most actions of lidocaine on the muscle-type nAChR can be ascertained by using structural analogs of either its hydrophilic (diethylamine; DEA) or hydrophobic (dimethylaniline; DMA) moieties (Alberola-Die et al., 2016a,b). The polar, charged DEA is responsible for the voltage-dependent blockade of nAChRs. DEA also elicits closed-channel blockade, mainly through its action on residues at the extracellular domain (ECD) (Alberola-Die et al., 2016a). In contrast, nAChR blockade by the uncharged, hydrophobic DMA is voltage-independent (although it can bind to the open-channel pore), and it mainly occurs through interactions outside the pore both at the ECD and, preferentially, at inter-subunit crevices on the transmembrane-spanning domain (TMD) to elicit closed-channel blockade. Moreover, DMA enhances nAChR desensitization (Alberola-Die et al., 2016b).

**Figure 1:**
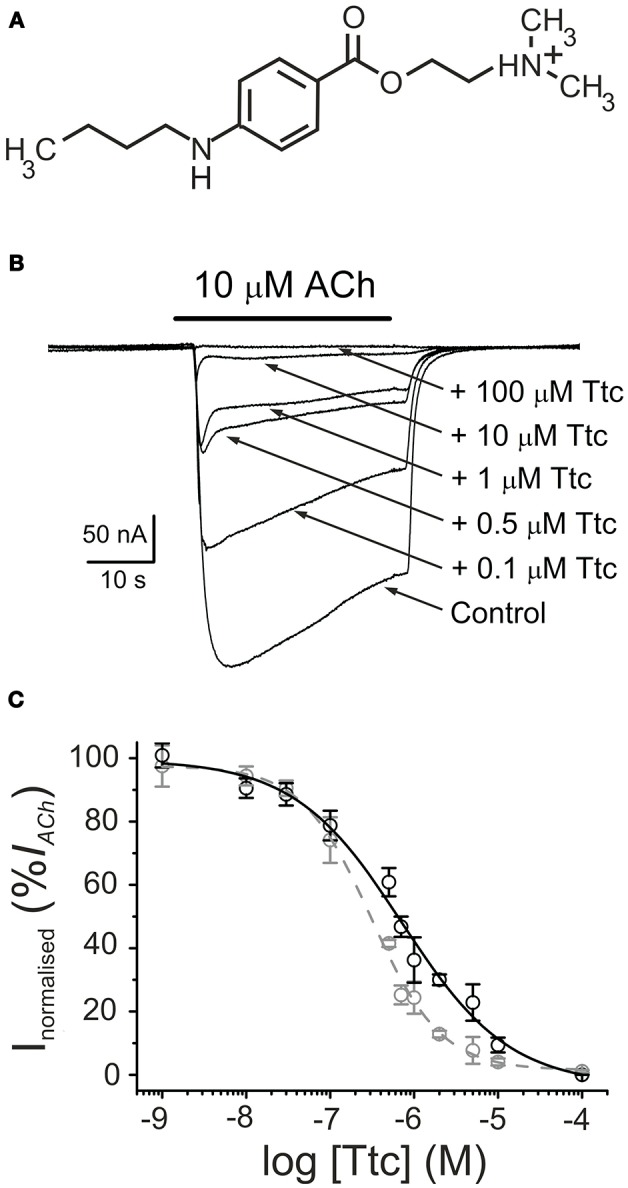
Tetracaine (Ttc) inhibition of currents elicited by ACh (*I*_*ACh*_s). **(A)** Molecular structure of Ttc, showing the amine group largely charged at the recording pH. **(B)** Superimposed *I*_*ACh*_*s* elicited by 10 μM ACh either alone (Control) or co-applied with different Ttc concentrations, as stated on the right. Note that *I*_*ACh*_decay was accelerated at Ttc concentrations of 0.5 μM or higher. Unless otherwise stated, the holding potential was −60 mV, downward deflections represent inward currents and the bars above the recordings indicate the timing of drug application. **(C)** Ttc concentration-*I*_*ACh*_ inhibition relationship. Peak (*I*_*p*_; black symbols) and steady state (*I*_*ss*_, measured 20 s after the peak; gray symbols) *I*_*ACh*_ amplitudes elicited in the presence of Ttc were normalized to the *I*_*ACh*_ evoked by ACh alone (Control) and represented against the logarithm of Ttc concentration. Solid and dashed lines are sigmoid curves fitted to *I*_*p*_ and *I*_*ss*_ data, respectively. Note that both curves overlap up to 0.1 μM Ttc. Error bars indicate SEM. Each point is the average of 4–23 oocytes from 3 to 11 frogs.

As a long-lasting amino-ester anesthetic, Ttc (2-(dimethylamino)ethyl 4-(butylamino)benzoate) is widely used in topical preparations, as well as spinal anesthesia and plexus/major nerve blocks, especially when a long duration of anesthesia is required. Similar to lidocaine, Ttc belongs to the group of LAs (Arias, 1999) that possess a single aromatic ring (see Figure 1A). However, it also has an ester group that is linked to an aliphatic chain that ends in a tertiary amine, which at pH 7, is largely protonated. Besides blocking voltage-dependent Na^+^ channels, Ttc has inhibitory effects on muscle (Gallagher and Cohen, 1999; Middleton et al., 1999) and neuronal nAChRs (Gentry and Lukas, 2001), as well as on high-voltage-activated calcium channels (Sugiyama and Muteki, 1994), ryanodine receptors (Zucchi and Ronca-Testoni, 1997), and acid-sensing ion channels (ASICs) (Leng et al., 2013). However, the mechanisms underlying the effects of Ttc on nAChRs remain largely unknown. Since some LAs with amine groups act on nAChRs by different mechanisms and the heterogeneity of their actions are, at least partially, related to the equilibrium between charged and uncharged forms, we have now explored the mechanisms of nAChR blockade by Ttc. Considering its pK_a_ of 8.4 (Chemicalize, https://chemicalize.com/), almost 97% of Ttc molecules are in a charged form at the recording pH (pH = 7.0). Notably, we found that Ttc induced a much stronger blockade of muscle-type nAChRs than either lidocaine or its charged hydrophilic moiety, DEA. We have now discovered that a roughly homogeneous pool of Ttc molecules elicit both open- and closed-channel blockade and markedly increase nAChR desensitization. These heterogeneous effects of Ttc on nAChRs are mediated by its interaction with different nAChR residues, located at both the ECD and the TMD.

Preliminary results have been published elsewhere in an abstract form (Cobo et al., 2014).

## Materials and methods

### Purification and reconstitution of nAChRs

The nAChRs from *Torpedo marmorata* electroplaques were purified by bromoacetylcholine-affinity chromatography in the presence of asolectin lipids, using cholate as a detergent. After elution with carbamylcholine, purified receptors were dialyzed and reconstituted in asolectin lipids at a final protein concentration of 0.3–1.2 mg/mL. Samples were aliquoted and stored in liquid nitrogen (Ivorra et al., 2002).

### Oocyte preparation and microinjection

Adult female *Xenopus laevis* (purchased from Harlan Interfauna Ibérica S.L., Barcelona, Spain; and Centre National de la Recherche Scientifique, Montpellier, France) were immersed in cold 0.17% tricaine methanesulfonate (MS-222) for 20 min, and a piece of the ovary was drawn out aseptically. Animal handling was carried out in accordance with the guidelines for the care and use of experimental animals adopted by the European Union, and the animal protocol was approved by the Ethics Committee of Universidad de Alicante. Stage V and VI oocytes were isolated and their surrounding layers were removed manually. Cells were kept at 15–16°C in a modified Barth's solution (88 mM NaCl, 1 mM KCl, 2.40 mM NaHCO_3_, 0.33 mM Ca(NO_3_)_2_, 0.41 mM CaCl_2_, 0.82 mM MgSO_4_, 10 mM 2-[4-(2-hydroxyethyl)piperazin-1-yl]ethane-1-sulfonic acid (HEPES; pH 7.4), 100 U/mL penicillin, and 0.1 mg/mL streptomycin) until further use. Oocytes were microinjected with 100 nL of an aliquot of reconstituted nAChRs (Morales et al., 1995).

### Two-electrode voltage-clamp recordings in oocytes

Membrane current recordings were performed at 21–25°C, 16–72 h after injection of proteoliposomes, using a high-compliance two-microelectrode voltage-clamp system (TurboTEC-10CD, npi Tamm, Germany). The recording methodology has been previously described (Morales et al., 1995; Alberola-Die et al., 2016b). Briefly, oocytes were placed in a 150-μL recording chamber and continuously superfused with normal frog Ringer's solution (NR: 115 mM NaCl, 2 mM KCl, 1.8 mM CaCl_2_, 5 mM HEPES, pH 7.0) supplemented with 0.5 μM atropine sulfate (normal Ringer with atropine, ANR) to block any muscarinic response (Kusano et al., 1982). The membrane potential was held at −60 mV, unless otherwise specified. Oocytes were superfused with ACh and the other drugs under investigation that had been diluted in ANR solution. Superfusion of the oocytes was conducted at a flow rate of 13–17 mL/min. Membrane currents elicited by ACh (*I*_*ACh*_), either alone or co-applied with Ttc, were low-pass filtered at 30–1,000 Hz, after sampling at fivefold the filter frequency (Digidata series 1550 and 1440 A; Axon Instruments, Foster City, CA, USA), as recorded on two PC-computers using the WCP v. 4.8.6. package developed by J. Dempster (Strathclyde Electrophysiology Software, University of Strathclyde, Scotland, UK) and AxoScope v. 10.0.0.60 software (Molecular Devices Corporation, Sunnyvale, CA, USA).

### Experimental design

Experimental procedures were similar to those used to study the effects of lidocaine (Alberola-Die et al., 2011) and other modulators (Alberola-Die et al., 2016a,b) on nAChRs. Briefly, the Ttc concentration-*I*_*ACh*_ inhibition relationship was determined by measuring *I*_*ACh*_s evoked by 10 μM ACh alone, or together with different concentrations of Ttc. For the competition assays, ACh concentration-*I*_*ACh*_ amplitude curves were obtained by bathing injected oocytes with increasing concentrations of ACh, either alone or together with 0.7 μM Ttc. The *I*_*ACh*_s were normalized to the maximum *I*_*ACh*_ evoked by ACh alone, and the values were fitted to a sigmoid curve (see Equation 3 below). To allow nAChRs to recover from desensitization, the interval between consecutive ACh applications was at least 5 min. To assess the blockade of resting nAChRs by Ttc, we compared the *I*_*ACh*_s elicited by ACh (from 1 μM to 1 mM) alone, or co-applied with 0.7 μM Ttc, either directly, or after 12 s of Ttc pre-application (at the same concentration). To better characterize the effects of Ttc on nAChR desensitization and compare *I*_*ACh*_ deactivation in the presence and the absence of Ttc, in some experiments, the oocyte remained superfused with Ttc (at 0.1 or 0.7 μM) for 12 s after withdrawal of 100 μM ACh.

Voltage dependence of the *I*_*ACh*_ blockade by Ttc was assessed by: (i) applying a series of 800 ms voltage pulses (from −120 to +60 mV, in 20 mV steps) to the oocyte before ligand superfusion and during the *I*_*ACh*_ plateau elicited by 10 μM ACh, either alone, or co-applied with different concentrations of Ttc; the −120 mV pulse duration was extended up to 1500 ms to allow a more complete current relaxation. (ii) From a holding potential of −60 mV, applying a single 800 ms voltage pulse to either +40 or +60 mV during the *I*_*ACh*_ plateau elicited by 10 μM ACh, either alone, or when co-applied with 0.1 or 0.7 μM Ttc. (iii) Comparing the *I*_*ACh*_ blockade induced by co-application of 0.7 μM Ttc with 10 μM ACh, to the effect when 0.7 μM Ttc was just pre-applied or administered with a combined pre- and co-application, while holding the membrane potential either at −60 or +40 mV.

### Data analysis and statistical procedures

Inhibition curves were determined by measuring the *I*_*ACh*_ evoked by 10 μM ACh in the presence of different concentrations of Ttc. The *I*_*ACh*_s (both at the peak and 20 s after) elicited in the presence of Ttc were normalized to the *I*_*ACh*_ evoked by ACh alone. Data were fitted to a logistic curve with the Origin 6.1 software (OriginLab Corp. Northampton, MA, U.S.A.), using the following Equation (1):

$$\begin{array}{l} I_{ACh+Ttc}=\left(\;\frac{I_{ACh}max\;-I_{ACh}min}{1+\;{\left(\left[Ttc\right]/IC_{\mathrm{50}}\right)}^{n_{H}}}\right)+\;I_{ACh}min \end{array}$$

where *I*_*ACh*+*Ttc*_ is the *I*_*ACh*_ amplitude elicited by co-application of 10 μM ACh with Ttc at a given concentration ([Ttc]); *I*_*ACh*_*max* and *I*_*ACh*_*min* are the maximum and minimum *I*_*ACh*_s recorded, respectively; *IC*_*50*_ is the Ttc concentration required to inhibit half the maximum *I*_*ACh*_; and *n*_*H*_ is the Hill coefficient.

The rate of desensitization was determined from the *I*_*ACh*_ decay elicited by ACh (10 or 100 μM), either alone, or co-applied with different concentrations of Ttc (0.1–2 μM). The time constant of the *I*_*ACh*_ decay was obtained through fitting to an exponential decay curve using the OriginPro 8 software (OriginLab Corp. Northampton, MA, U.S.A.). In addition, based on the methods of Sobolevsky et al. (1999), the change in the rate of desensitization induced by Ttc (0.01–2 μM) was determined using the following Equation (2):

$$\begin{array}{l} I_{ACh}\;desensitization\;change=\left(\frac{I_{ss\text{\_}Ttc}/I_{p\text{\_}Ttc}}{I_{ss\text{\_}Ctr}/I_{p\text{\_}Ctr}}\right) \end{array}$$

where *I*_*p*_*Ctr*_ and *I*_*p*_*Ttc*_ are the *I*_*ACh*_ peaks elicited by ACh (10 or 100 μM) either alone, or together with Ttc, respectively; *I*_*ss*_*Ctr*_ and *I*_*ss*_*Ttc*_ are *I*_*ACh*_*s* 20 s after the corresponding *I*_*ACh*_ peaks.

To characterize the pharmacological profile of Ttc, nAChRs were activated by different concentrations of ACh alone, or co-applied with Ttc, at roughly its *IC*_*50*_, either directly, or after its pre-application for 12 s. Dose-response data were fitted to the following form of the Hill Equation (3):

$$\begin{array}{l} \frac{I}{I_{ACh}max}={\left[{1+\left(\frac{EC_{\mathrm{50}}}{\left[ACh\right]}\right)}^{n_{H}}\right]}^{-1} \end{array}$$

where *I* is the *I*_*ACh*_ amplitude elicited at a given concentration of ACh ([ACh]) applied either alone, or together with Ttc; *EC*_*50*_ is the agonist concentration required to obtain one-half the maximum *I*_*ACh*_; and *I*_*ACh*_*max* and *n*_*H*_ are as in Equation (1).

Net *i/v* curves for *I*_*ACh*_ were obtained by subtracting, for each voltage, the steady state currents attained in ANR (measured during the last 100 ms of the pulse) from the corresponding currents recorded in the presence of 10 μM ACh alone, or together with Ttc. These net *I*_*ACh*_ values were normalized, for each oocyte, to the ACh response at −60 mV.

Unless otherwise specified, values presented were the mean ± standard error of the mean (SEM); “*n*” indicates the number of oocytes and “*N*” is the number of oocyte-donor frogs from which the data were obtained. When comparing two-group means of normally distributed values, the Student's *t*-test was used; otherwise, the Mann–Whitney rank-sum test was applied. Among-group differences were determined by the analysis of variance (ANOVA), and mean differences for each pair of groups were determined with the Bonferroni *t*-test. The one-sample *t*-test was used to compare the mean of an experimental group with a specified value. For the comparison of *EC*_*50*_ and *IC*_*50*_ values, we used the confidence intervals (CIs) computed by the curve-fitting function of the Origin 6.1 software, using 95% confidence levels. The criterion of “non-overlapping 95% confidence intervals” was used to determine significant differences. A significance level of *p* < 0.05 was considered in all cases.

### Virtual docking assays

Docking assays were carried out as previously described (Alberola-Die et al., 2016a,b). Briefly, *Torpedo* nAChR structures in the closed (4 Å resolution, pdb code 2BG9; Unwin, 2005) and open (6.2 Å resolution, pdb code 4AQ9; Unwin, 1995; Unwin and Fujiyoshi, 2012), were obtained from the Research Collaboratory for Structural Bioinformatics (RCSB) Protein Data Bank (PDB). The specific edition of the protein was made using DeepView v4.1 (Guex and Peitsch, 1997) and Yasara (Krieger et al., 2002, 2004) software without further optimization. The structure of Ttc was obtained from the National Center for Biotechnology Information (NCBI) PubChem database (http://www.ncbi.nlm.nih.gov/pccompound). A global docking procedure was accomplished with AutoDock 4 (Morris et al., 2008) implemented in Yasara, in which a total of 800 flexible docking runs were set and clustered around the putative binding sites. The program then performed a simulated annealing minimization of the complexes, which moved the structure to a nearby stable energy minimum, by using the implemented (Assisted Model Building with Energy Refinement) AMBER 99 force field (Duan et al., 2003). The Yasara pH command was set to 7.0, to ensure that molecules preserved their pH dependency of bond orders and protonation patterns. In this way, 97% of the Ttc molecules were protonated. The best binding energy complex in each cluster was stored, analyzed, and used to select the best orientation of the interacting partners.

Global docking of Ttc on the nAChR channel pore systematically occurred at a single, high-affinity binding site. Thus, no other sites were found, following this strategy. In order to explore alternative binding sites with lower affinities within the pore, the high-affinity site was blocked with a Ttc molecule before starting subsequent docking runs. We used the best position of Ttc bound to the deep residues within the pore. In this way, we ensured that the high-affinity site was already occupied, and simulated a scenario with a high concentration of Ttc. Figures were drawn with open source PyMol (The PyMOL Molecular Graphics System, Version 1.8 Schrödinger, LLC, at http://www.pymol.org/).

The theoretical affinities of Ttc at its binding site can be determined by calculating the binding energy of the ligand-receptor complex. The binding energy is obtained by measuring the energy at infinite distance (the unbound state) and subtracting from that value the energy of the complex (the bound state). The relationship between the Gibbs free energy of binding (Δ*G*, cal/mol) and the dissociation constant (K_d_) was determined by the following Equation (4):

$$\begin{array}{l} \Delta G=\;-\;RT\ln\;K_{d} \end{array}$$

where *R* = 1.98 cal/molK and *T* = 298 K. Thus, the more positive the binding energy, the more favorable the interaction in the context of the chosen force field.

### Drugs

The drugs ACh, atropine sulfate, Ttc, MS-222, penicillin, and streptomycin were obtained from Sigma (St. Louis, MO, USA), and HEPES was obtained from Acros Organics (New Jersey, NJ, USA). The reagents of general use were purchased from Scharlau Chemie SA (Barcelona, Spain). All solutions were made in ANR just before each application.

## Results

### Inhibition of *I_*Ach*_* by Ttc

The superfusion of Ttc (see chemical structure in Figure 1A) on either uninjected oocytes or those bearing nAChRs, with the membrane potential clamped at −60 mV, did not modify their cell membrane conductance, even at concentrations as high as 1 mM. In contrast, co-application of 10 μM ACh with 1 nM−100 μM Ttc reversibly inhibited *I*_*ACh*_, in a dose-dependent manner (Figure 1B). The *IC*_*50*_ and *n*_*H*_ values (see Equation 1) for the *I*_*ACh*_ peak (*I*_*p*_) were 0.7 μM (95% CI, 0.5–0.9 μM; *n* = 4–23, *N* = 3–11) and 0.7 ± 0.1, respectively (black circles and continuous line; Figure 1C). At low Ttc concentrations (up to 0.1 μM), this *I*_*p*_ inhibition was similar to that measured 20 s after *I*_*p*_, which will be referred to hereafter as the “steady state current” (*I*_*ss*_). However, at Ttc concentrations higher than 0.1 μM, the *I*_*ss*_ blockade was significantly greater than the corresponding *I*_*p*_ inhibition (see Figure 1B). Thus, the dose-inhibition curve for the *I*_*ss*_ showed an *IC*_*50*_ of 0.3 μM (95% CI, 0.2–0.4 μM, same cells and donor frogs as above) and a slope of 1.0 ± 0.1 (gray circles and dashed line; Figure 1C). Interestingly, the effects of Ttc on muscle-type nAChR was specific, as gamma-aminobutyric acid (GABA_A_) receptors (GABA_A_Rs), which also belong to the Cys-loop family of receptors, were not noticeably affected by Ttc, even at concentrations of 100-fold the *IC*_*50*_ of Ttc on *I*_*ACh*_ (see Figure S1).

### Voltage dependence of nAChR blockade by Ttc

**Figure 2:**
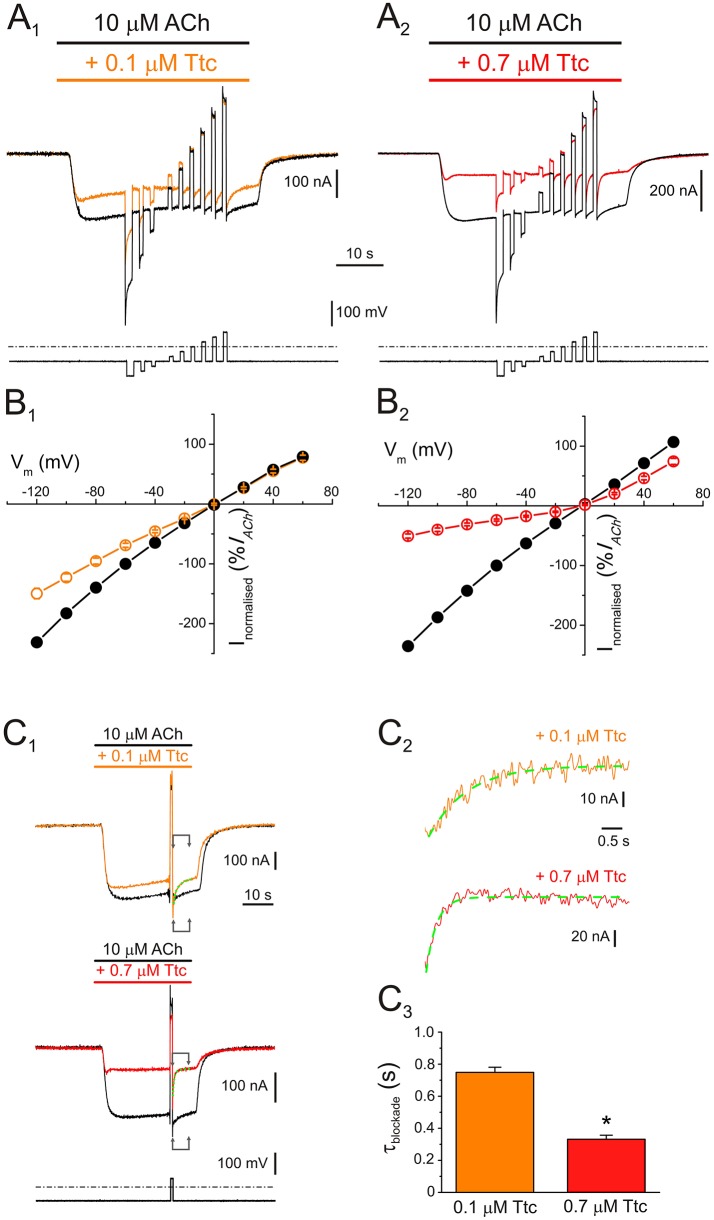
Voltage dependence of nicotinic acetylcholine receptor (nAChR) blockade by tetracaine (Ttc). **(A)** *I*_*ACh*_s (upper traces) elicited by 10 μM ACh either alone (**A**_1_,**A**_2_, black recordings), or in the presence of 0.1 μM (**A**_1_, orange), or 0.7 μM Ttc (**A**_2_, red) when the voltage protocol, indicated below the currents, was applied. **(B)** Plots of net *i/v* relationships for *I*_*ACh*_s evoked, following the protocol shown in **A**. Control *I*_*ACh*_s are represented by black symbols and lines **(B**_1_**,B**_2_**)**, whereas those evoked in the presence of 0.1 μM (**B**_1_) and 0.7 μM Ttc (**B**_2_) are drawn in orange and red, respectively. Values were normalized as a percentage of current with reference to their control *I*_*ACh*_ at −60 mV. Each point is the average of 5 (*N* = 1) and 12 (*N* = 3) cells for 0.1 and 0.7 μM Ttc, respectively. **(C)** Kinetics of the voltage-dependent blockade of nAChRs at −60 mV. **(C**_1_**)** *I*_*ACh*_s were elicited by 10 μM ACh alone (control, black recordings), or together with either 0.1 μM (orange trace) or 0.7 μM Ttc (red recording) at −60 mV; during the *I*_*ACh*_ plateau, an 800 ms voltage jump to +40 mV was given (bottom trace shows the voltage protocol). Membrane leak currents (recorded in the absence of ACh) have been subtracted. **(C**_2_**)** Zoomed in view of the areas indicated by arrows in **C**_1_ (immediately after the voltage jump). Kinetics of the voltage-dependent blockade of nAChRs by 0.1 μM (orange trace) and 0.7 μM Ttc (red trace) were determined by fitting the net *I*_*ACh*_ decays to exponential functions (green curves over the recordings). The small, slow *I*_*ACh*_ changes evoked by the voltage pulse when the cell was bathed solely with ACh (black recordings in **C**_1_) have been subtracted. **(C**_3_**)** Time constant values of the voltage-dependent *I*_*ACh*_ blockade kinetics elicited by 0.1 and 0.7 μM Ttc. Asterisk indicates significant differences between both values (*p* < 0.05, *t*-test).

To elucidate whether *I*_*ACh*_ inhibition by Ttc is voltage-dependent, voltage pulses were applied to oocytes while superfusing them with ANR, or during the *I*_*ACh*_ plateau elicited by 10 μM ACh, either alone, or together with 0.1 or 0.7 μM Ttc (Figures 2A_1_,A_2_, respectively; see Experimental design in Material and methods). The *i/v* curves of net *I*_*ACh*_*s* elicited by ACh, either alone, or co-applied with 0.1 or 0.7 μM Ttc, showed that neither *I*_*ACh*_ reversal potential, close to 0 mV, nor its inward rectification were affected by the presence of Ttc (Figures 2B_1_,B_2_; see also Morales et al., 1995). However, co-application of 10 μM ACh with 0.1 μM Ttc reduced *I*_*ACh*_ amplitude in a voltage-dependent manner; thus, the more hyperpolarized the membrane potential, the larger the blockade (Figure 2B_1_). This suggests that Ttc, at this concentration, mainly binds within the channel pore.

An additional mechanism of blockade was present when 0.7 μM Ttc was co-applied with ACh, which roughly accounted for 35% of *I*_*ACh*_ inhibition at positive potentials (Figure 2B_2_). It ought to be considered that positive potentials should eject the positively charged Ttc from the channel pore. Therefore, this added *I*_*ACh*_ inhibition seems voltage-independent, indicating that at concentrations close to the *IC*_*50*_, Ttc also interacts with nAChR residues located outside the pore. Nonetheless, the voltage-dependent blockade of *I*_*ACh*_ remained at 0.7 μM Ttc (compare the extent of *I*_*ACh*_ blockade at negative *versus* positive potentials in Figure 2B_2_; see also Figure S2, which plots the *I*_*ACh*_ remnant vs. membrane potential when ACh was co-applied with different concentrations of Ttc). This voltage-dependent inhibition facilitates the estimation of the apparent rate of channel pore blockade. Thus, during the *I*_*ACh*_ plateau elicited by 10 μM ACh in the presence of 0.1 or 0.7 μM Ttc, the membrane potential was stepped back to −60 mV, after an 800 ms pulse at either +40 or +60 mV, (Figure 2C_1_). As shown in Figure 2C_2_, *I*_*ACh*_ blockade at −60 mV followed an exponential function with time constant values of 749 ± 32 ms (*n* = 10, *N* = 3) and 332 ± 25 ms (*n* = 11; *N* = 4) for 0.1 and 0.7 μM Ttc, respectively. Thus, the kinetics of the voltage-dependent blockade of *I*_*ACh*_ were accelerated with increasing Ttc concentration (*p* < 0.05, *t*-test; Figure 2C_3_).

### Pharmacological profile of *I_*ACh*_* blockade by Ttc

**Figure 3:**
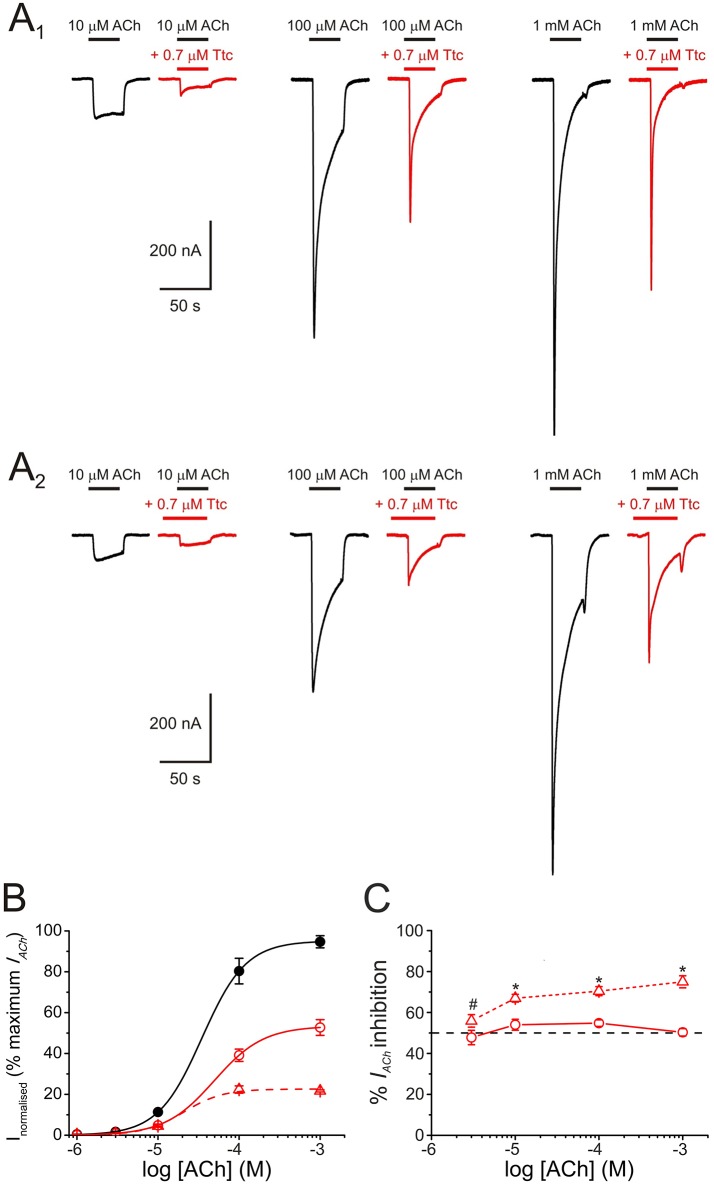
Pharmacological profile of nicotinic acetylcholine receptor (nAChR) blockade by tetracaine (Ttc). **(A)** *I*_*ACh*_s evoked by different ACh concentrations (10, 100 μM, and 1 mM) either alone (**A**_1_,**A**_2_, black recordings), co-applied with 0.7 μM Ttc (**A**_1_, red recordings), or co-applied with 0.7 μM Ttc, after Ttc pre-application for 12 s at the same concentration (**A**_2_, red recordings). **(B)** Averaged ACh concentration-*I*_*ACh*_amplitude relationship. *I*_*ACh*_s were evoked by different ACh concentrations alone (filled black circles; *n* = 10–13, *N* = 3), or co-applied with 0.7 μM Ttc, either directly (open circles; *n* = 3–6, *N* = 2), or subsequent to its pre-application (open triangles; *n* = 4–7, *N* = 2). Data were normalized to the maximal *I*_*ACh*_ elicited by ACh alone and fitted to the Hill equation (solid and dashed lines). **(C)** Percentage of *I*_*ACh*_ inhibition when different ACh concentrations were directly co-applied with 0.7 μM Ttc (circles and solid line; *n* = 9–33, *N* = 4–13), or after pre-application of the same Ttc concentration for 12 s (triangles and dashed line; *n* = 11–21, *N* = 2–6). Asterisks indicate significant differences in the percentage of *I*_*ACh*_ inhibition between ACh-Ttc co-application alone, and pre- and co-application of Ttc at each ACh concentration (*p* < 0.05, *t*-test). ACh concentration effected no significant changes in the extent of inhibition by Ttc, either when Ttc and ACh were directly co-applied, or when this co-application was preceded by Ttc pre-application (*p* > 0.05, ANOVA; except at 3 μM ACh, indicated by the pound sign. However, *I*_*ACh*_s at such low ACh concentration are too small for accurate determination of the percentage of inhibition).

To better characterize the mechanisms underlying *I*_*ACh*_ inhibition by Ttc, oocytes were superfused with ACh at different concentrations (1, 3, 10, 100 μM, and 1 mM) alone, or co-applied with 0.7 μM Ttc, either directly, or after a 12 s pre-application of the same Ttc concentration (Figure 3A_1_,A_2_, respectively). When ACh and Ttc were directly co-applied, the *I*_*ACh*_ amplitude was reduced roughly by half (as would be expected from the estimated *IC*_*50*_ of Ttc), independently of the ACh dose tested (see records in Figures 3A_1_,B,C). This indicates that Ttc was acting by a non-competitive mechanism of inhibition. The estimated *EC*_*50*_ values of the ACh dose-*I*_*ACh*_ amplitude curves obtained when Ttc was either co-applied, or pre- and co-applied with ACh, were similar to those observed in control curves, in which ACh was applied alone. In particular, the *EC*_*50*_ values were 37 μM for the control curve (95% CI 32–43 μM; *n* = 10–13, *N* = 3) vs. 49 μM (95% CI 42–55 μM; *n* = 3–6, *N* = 2) and 21 μM (95% CI 12–36; *n* = 4–7, *N* = 2) for the sole Ttc and ACh co-application, and the Ttc pre- and co-application, respectively (*p* > 0.05). The *I*_*ACh*_ blockade elicited by pre- and co-application of Ttc was also independent of the ACh dose (56–75% at different concentrations). However, the extent of *I*_*ACh*_ inhibition was significantly greater than that observed with ACh and Ttc co-application alone (48–55%, *p* < 0.05 ANOVA; Figure 3C), unless at very low ACh concentrations (3 μM). Since the probability of unliganded nAChRs being open is less than one in a million (Nayak et al., 2012), it turns out that the increased nAChR blockade by Ttc pre-application, before its co-application with ACh, is due to Ttc binding to resting (closed) nAChRs. Consequently, Ttc pre-application would block nAChRs before they can be gated by the agonist.

### Differential effects of Ttc on *I_*ACh*_* depending on membrane potential and application time

**Figure 4:**
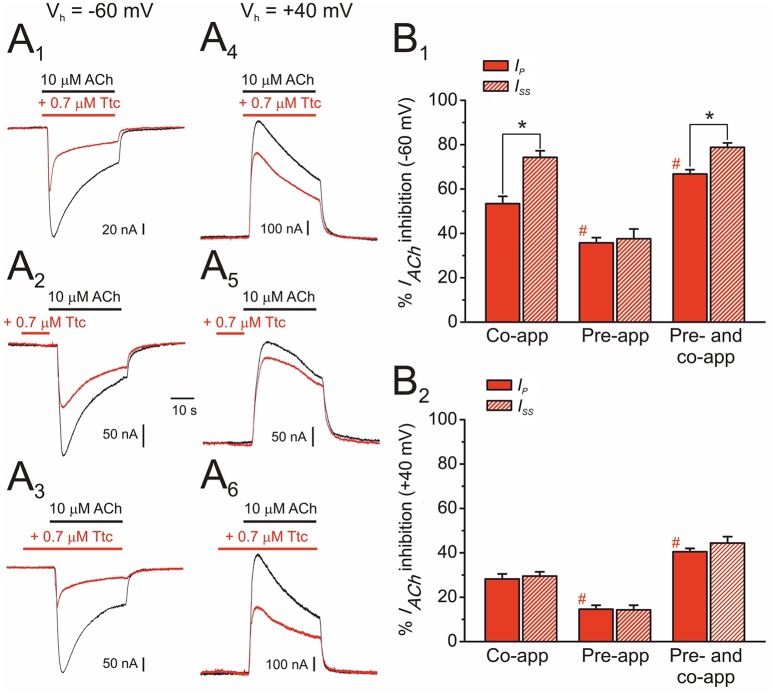
Effect of tetracaine (Ttc) application timing and holding potential on nicotinic acetylcholine receptor (nAChR) blockade. **(A)** *I*_*ACh*_*s* elicited at −60 mV **(A**_1_**,A**_2_**,A**_3_**)** and at +40 mV **(A**_4_**,A**_5_**,A**_6_**)** by co-application of 10 μM ACh and 0.7 μM Ttc (Co-app; **A**_1_**,A**_4_), sole Ttc pre-application before superfusion of the agonist (Pre-app; **A**_2_**,A**_5_), or Ttc pre-application followed by its co-application with ACh (Pre- and co-app; **A**_3_**,A**_6_). **(B)** Column graphs showing the percentages of *I*_*ACh*_ inhibition by Ttc at −60 mV **(B**_1_**)** and +40 mV **(B**_2_**)** at the *I*_*p*_ (red filled columns) and the *I*_*ss*_ (red striped columns), when Ttc was applied as indicated in **(A)**. Asterisks indicate significant differences between *I*_*ACh*_ inhibition by Ttc at *I*_*p*_ and *I*_*ss*_ (*p* < 0.05, paired *t*-test). Pound signs indicate significant differences of *I*_*p*_ inhibition among Ttc application-timing protocols, as compared with the values for ACh and Ttc co-application (*p* < 0.05, ANOVA and Bonferroni *t*-test). Note that *I*_*ACh*_ decay was only accelerated (i.e., significant differences observed between *I*_*p*_ and *I*_*ss*_ inhibition) when Ttc was either co-applied with ACh, or pre-applied and later co-applied with ACh, at −60 mV. Each column represents the average obtained from 12 to 20 oocytes (*N* = 4–9) for **(B**_1_**)**, and from 6 to 11 cells (*N* = 2–3) for **(B**_2_**)**.

As mentioned above, nAChR inhibition by Ttc at relatively high concentrations (*IC*_*50*_ or above) involved both open- and closed-channel blockade. To better understand the effects of Ttc on nAChRs at these concentrations, oocytes were clamped at two different potentials (−60 or +40 mV), and *I*_*ACh*_*s* were elicited by 32 s superfusion of 10 μM ACh either alone (Figures 4A_1_-A_6_, black recordings), or with 0.7 μM Ttc (Figures 4A_1_-A_6_, red recordings) in three different protocols as follows: (i) Ttc was co-applied with ACh (Figures 4A_1_,A_4_); (ii) Ttc was pre-applied for 12 s before superfusion with ACh alone (Figures 4A_2_,A_5_); and (iii) 12 s pre-application of Ttc followed by its co-application with ACh (Figures 4A_3_,A_6_). The percentages of *I*_*p*_ and *I*_*ss*_ inhibition by Ttc differed, depending on the specific protocol (Figures 4B_1_,B_2_). Thus, in oocytes clamped at −60 mV, co-application of Ttc and ACh blocked roughly half the control *I*_*p*_, as expected from its estimated *IC*_*50*_ (53 ± 3%, *n* = 20, *N* = 9; Figure 4B_1_). However, the percentage of *I*_*ss*_inhibition increased to 74 ± 3% (same cells; *p* < 0.05, paired *t*-test; Figure 4B_1_), mainly because of the acceleration of *I*_*ACh*_ decay (compare black and red recordings of Figure 4A_1_). When Ttc was solely pre-applied, before superfusion with ACh alone, the percentage of *I*_*p*_ inhibition was significantly less (36 ± 2%; *n* = 12, *N* = 4; *p* < 0.05, ANOVA; Figure 4B_2_) than when Ttc and ACh were co-applied. No significant differences were noted between the percentages of *I*_*p*_ and *I*_*ss*_inhibition when Ttc was solely pre-applied (Figure 4B_1_), indicating a very slow recovery from this blockade. In contrast, Ttc pre-application, followed by its co-application with ACh significantly increased the percentage of *I*_*p*_ inhibition, as compared with their sole co-application (67 ± 2% vs. 53 ± 3%; *n* = 15, *N* = 5; *p* < 0.05, ANOVA and Bonferroni *t*-test; Figure 4B_1_). Furthermore, the *I*_*ss*_ blockade (79 ± 2%) was significantly greater than the *I*_*p*_ inhibition (*p* < 0.05, paired *t*-test), indicating that *I*_*ACh*_ decay was accelerated when Ttc was pre-applied, and later co-applied with ACh. Indeed, the *I*_*ACh*_ decay time constants (see below) observed when Ttc was co-applied with ACh alone, and when Ttc was pre-applied and then co-applied with ACh were similar (compare Figure 4A_1_ and Figure 4A_3_). It is also interesting that the percentage of *I*_*p*_ remnant when Ttc was first pre-applied, and then co-applied with ACh (33%) was very close to the expected value if Ttc binding sites with sole Ttc pre-application, and Ttc and ACh co-application (64 and 47% of control *I*_*p*_, respectively) were independent (30%). Thus, interactions of Ttc with resting and open nAChRs agree with an allotopic model, as we previously observed for DMA and DEA interactions on nAChRs (Alberola-Die et al., 2016b).

When the membrane potential was held at +40 mV, the extent of *I*_*ACh*_ blockade by 0.7 μM Ttc was smaller than that at −60 mV in any of the three above-mentioned protocols (Figure 4A_4_-A_6_). Nevertheless, as observed at −60 mV, the highest *I*_*ACh*_ inhibition was found with Ttc pre- and co-application, and the lowest inhibition with just Ttc pre-application (Figure 4B_2_). Interestingly, at +40 mV, *I*_*ACh*_ decay was not enhanced by Ttc when it was either just co-applied with ACh or pre- and co-applied, in contrast to its effects observed at −60 mV. Therefore, at positive potentials the percentages of *I*_*p*_ and *I*_*ss*_ inhibition were similar in all tested protocols (Figure 4B_2_).

**Figure 5:**
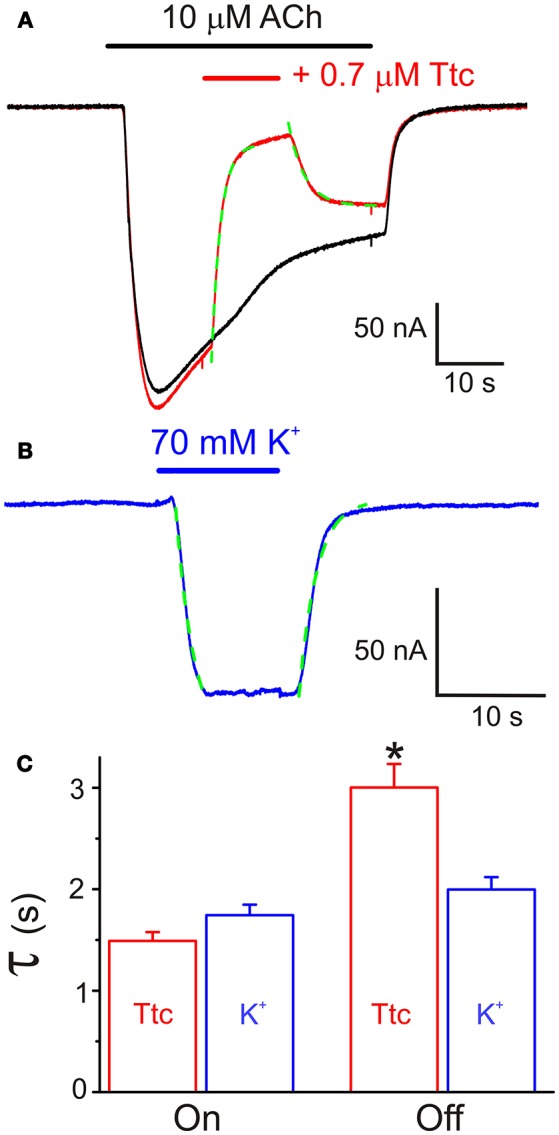
Effect of tetracaine (Ttc) application while nicotinic acetylcholine receptors (nAChRs) were activated by 10 μM acetylcholine (ACh). **(A)** Two superimposed *I*_*ACh*_*s* elicited by 40 s pulses of ACh. The red recording shows the fast inhibitory effect of 0.7 μM Ttc, superfused when indicated by the red horizontal bar. The kinetics of *I*_*ACh*_ inhibition followed an exponential function (green trace) with a time constant similar to those found for membrane currents evoked by superfusion of a high-K^+^ (70 mM) solution (blue recording in **B**). Onset and decay of the K^+^ current were fitted to exponential functions (discontinuous green line). **(C)** Time constant values of the exponential functions fitted to the onset (On) and recovery (Off) of *I*_*ACh*_ blockade by Ttc and K^+^ currents. Note that the rate of *I*_*ACh*_ inhibition is conditioned by the solution exchange kinetics, but *I*_*ACh*_ recovery, after Ttc removal, exhibited slower kinetics. Asterisk indicates significant differences (*n* = 13, *p* < 0.05, ANOVA test).

In addition, we tested the effects of a 12 s pulse of 0.7 μM Ttc, applied during the *I*_*ss*_ elicited by a 40 s pulse of 10 μM ACh (Figure 5A). This co-application of ACh and Ttc evoked a fast and large inhibition of *I*_*ss*_ (*I*_*ss*_ reduced by 75 ± 2%; *n* = 13, *N* = 4). Notably, the kinetics of this *I*_*ACh*_ blockade showed the same temporal course as that observed for membrane currents elicited by superfusion with a high-K^+^ solution. This indicates that the timing of this *I*_*ss*_ inhibition was only limited by the perfusion kinetics (see Figures 5B,C). In contrast, *I*_*ss*_ recovery after Ttc removal exhibited slower kinetics (time constants of 1.49 ± 0.09 s vs. 3.00 ± 0.23 s, for Ttc onset and recovery phases, respectively; *p* < 0.05, *t*-test), which was not limited by the solution exchange kinetics (compare with the high-K^+^ solution washout; Figure 5C). Nevertheless, the kinetics of *I*_*ACh*_ recovery when Ttc blocked open nAChRs was much faster than that after the blockade of closed nAChRs (see Figure 4A_2_).

### Ttc enhancement of nAChR desensitization

**Figure 6:**
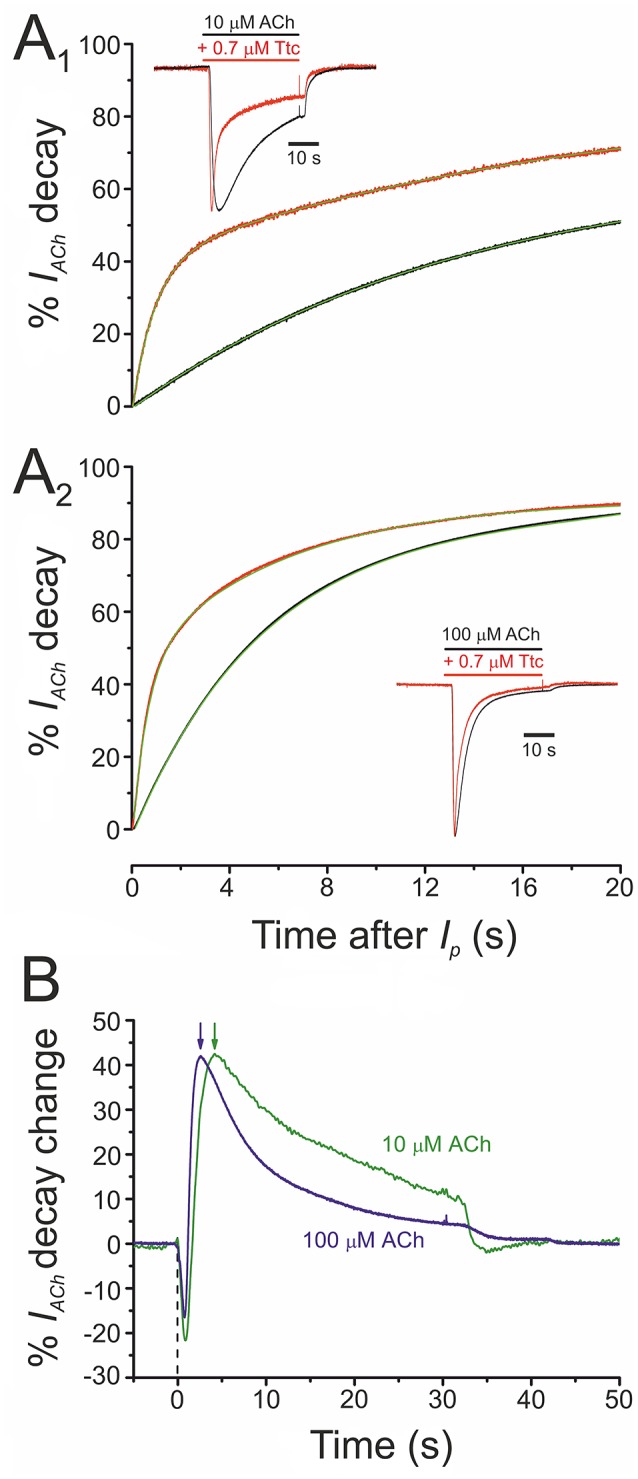
Acceleration of *I*_*ACh*_ decay by tetracaine (Ttc) is dependent on the concentration of acetylcholine (ACh). **(A)** Plot of averaged *I*_*ACh*_ decay (expressed in percentages) elicited by 10 μM ACh (**A**_1_; *n* = 12, *N* = 6) or 100 μM ACh (**A**_2_; *n* = 18, *N* = 6), either alone (black recordings) or co-applied with 0.7 μM Ttc (red traces). Green lines over the averaged recordings represent two-exponential functions fitted to the *I*_*ACh*_ decay. Insets are representative recordings of *I*_*ACh*_s elicited by 10 μM ACh **(A**_1_**)** or 100 μM ACh **(A**_2_**)**, either alone (black recordings), or co-applied with 0.7 μM Ttc (red traces). The *I*_*p*_ amplitudes in the presence of Ttc have been normalized to their control values for easier comparison of decay kinetics. **(B)** Averaged percentages of change in *I*_*ACh*_ decay elicited by 0.7 μM Ttc, computed as the difference between *I*_*ACh*_s obtained in the absence and presence of Ttc, for currents evoked by 10 μM ACh (green line; *n* = 12; *N* = 6) or 100 μM ACh (blue line; *n* = 18, *N* = 6). Notice the earlier maximum decay acceleration (arrows) when *I*_*ACh*_ was evoked by 100 μM ACh. Zero time corresponds to the beginning of Ttc-ACh co-application and the downward deflections are due to the earlier *I*_*p*_ in the presence of Ttc (see inset of **A**_1_).

At concentrations of 0.5 μM or higher, Ttc accelerated *I*_*ACh*_ decay (see Figure 1). This acceleration might have originated from one of the following two mechanisms (or a combination of both): (i) a slow blocking effect of Ttc on nAChRs, which would boost *I*_*ACh*_ decline after its peak; (ii) an enhancement of nAChR desensitization. To discriminate between both possibilities, we assessed the effect of 0.7 μM Ttc on the *I*_*ACh*_ decay elicited by two different concentrations of ACh (10 and 100 μM), because desensitization is markedly dependent on agonist concentration. As previously reported, *I*_*ACh*_ decay followed a two-exponential function (see Figures 6A_1_,A_2_), although the slower component was too slow for accurate analysis using this experimental approach (Morales and Sumikawa, 1992). Thus, considering only the time constant (τ) of the fast component of *I*_*ACh*_ decay, it is clear that *I*_*ACh*_ declined at a slower rate at 10 μM ACh (Figure 6A_1_; τ_Ctr_ = 15.6 ± 2.1 s, *n* = 12, *N* = 6) than at 100 μM ACh (Figure 6A_2_; τ_Ctr_ = 5.9 ± 0.7 s, *n* = 18, *N* = 6; *p* < 0.05, *t*-test). In the presence of 0.7 μM Ttc, the *I*_*ACh*_ decay showed a different trend in acceleration at 10 μM (τ_Ttc_ = 1.0 ± 0.1 s) and at 100 μM ACh (τ_Ttc_ = 0.6. ± 0.1 s; *p* < 0.05, *t*-test; Figures 6A_1_,A_2_). Thus, a constant Ttc dose had a more potent effect on accelerating *I*_*ACh*_ decay when a higher concentration of ACh was used. This finding rules out the notion that the enhancement of *I*_*ACh*_ decay is merely due to a delayed nAChR blockade, mediated by slow Ttc binding. Therefore, the maximum percentage of change in *I*_*ACh*_ decay elicited by 0.7 μM Ttc was achieved earlier, when it was co-applied with 100 μM ACh (2.6 s) than with 10 μM ACh (4.2 s; Figure 6B, arrows). These results strongly suggest that Ttc enhances nAChR desensitization.

**Figure 7:**
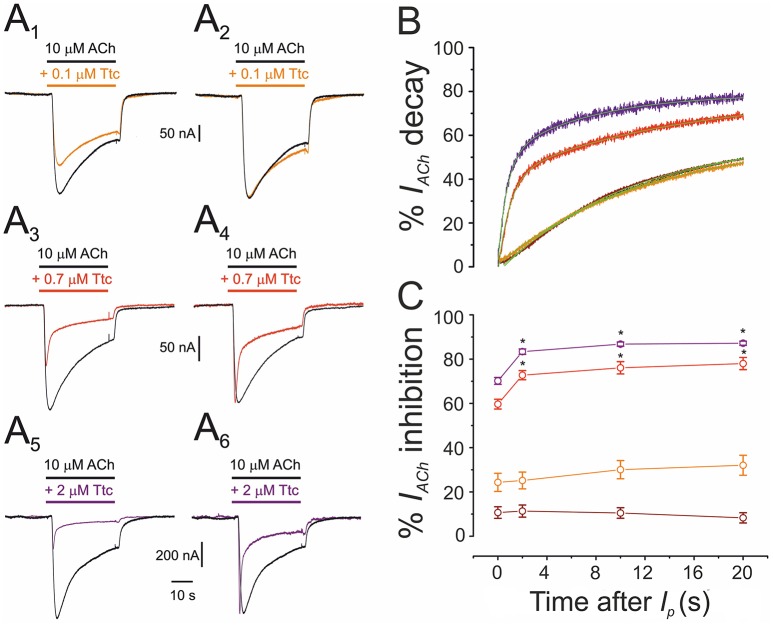
Acceleration of the decay of currents elicited by ACh (*I*_*ACh*_) is dependent on the concentration of tetracaine (Ttc). **(A)** Representative *I*_*ACh*_s elicited by 10 μM ACh, either alone (black; **A**_1_**-A**_6_), or co-applied with 0.1 μM (orange; **A**_1_), 0.7 μM (red; **A**_3_), or 2 μM Ttc (purple; **A**_5_). The same *I*_*ACh*_s are in the right-hand **(A**_2_**,A**_4_**,A**_6_**)**, but their peak amplitudes were normalized to show more effectively the differences on *I*_*ACh*_ decay. **(B)** Normalized and averaged *I*_*ACh*_ decay elicited by 10 μM ACh, either alone (black trace; *n* = 43, *N* = 12), or plus 0.01 μM (brown trace; *n* = 12, *N* = 4); 0.1 μM (orange trace; *n* = 9, *N* = 3); 0.7 μM (red trace; *n* = 12, *N* = 6); or 2 μM Ttc (purple trace; *n* = 10, *N* = 4). Each averaged *I*_*ACh*_ decay was fitted by a two-exponential function (green lines overlapping each recording). Note that with Ttc concentrations of up to 0.1 μM, the *I*_*ACh*_ decay overlaps the control. **(C)** Percentages of *I*_*ACh*_ inhibition by 0.01, 0.1, 0.7, and 2 μM Ttc (same cells and color codes as in **B**) at different times after *I*_*p*_. Low Ttc concentrations blocked nAChRs, but they did not modify *I*_*ACh*_ decay. In addition, note that the time-dependent increase in the percentage of *I*_*ACh*_ inhibition was already established 2 s after *I*_*p*_. For each Ttc concentration, asterisks indicate significant differences among the percentages of *I*_*ACh*_ inhibition at different times, as compared with their respective *I*_*p*_ (*p* < 0.05, ANOVA, Bonferroni *t*-test).

Co-application of 10 μM ACh with different concentrations of Ttc (0.01–2 μM) also highlights the fact that Ttc enhances nAChR desensitization at concentrations close to, or above its *IC*_*50*_. As shown in Figure 7, low Ttc concentrations (0.01–0.1 μM) elicited a significant *I*_*ACh*_ blockade (up to 30%; Figures 7A_1_,C), but did not modify *I*_*ACh*_ decay (Figures 7A_2_,B). In contrast, ACh co-applied with 0.7–2 μM Ttc significantly increased both the extent of nAChR blockade (Figures 7A_3_,A_5_,C) and the rate of *I*_*ACh*_ decay (Figures 7A_4_,A_6_,B). The lack of an acceleration of *I*_*ACh*_ decay by low Ttc concentrations, which nonetheless reduced *I*_*ACh*_ amplitude, presents evidence against the hypothesis of a slow nAChR blockade by Ttc being responsible for a boost in *I*_*ACh*_ decay, and supports the theory that Ttc indeed enhances nAChR desensitization.

**Figure 8:**
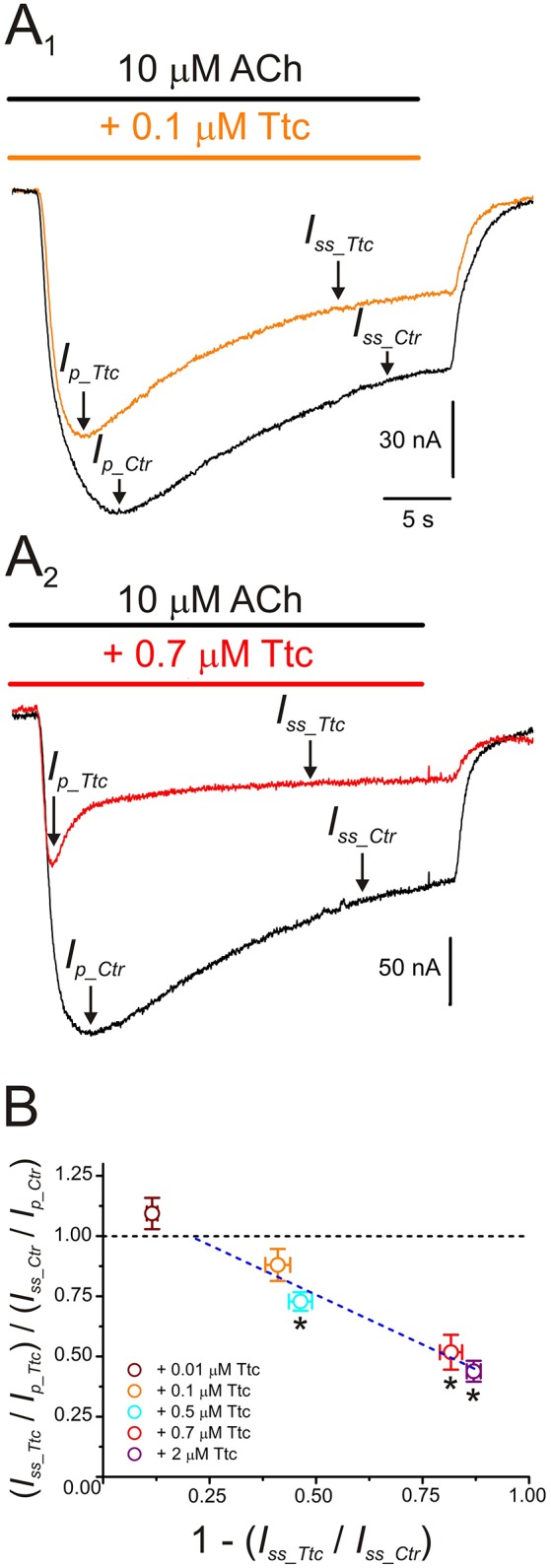
*I*_*ACh*_ desensitization increases with increasing tetracaine (Ttc) concentration. **(A)** *I*_*ACh*_s evoked by 10 μM ACh, either alone (black; **A**_1_,**A**_2_) or co-applied with 0.1 μM (orange recording; **A**_1_), or 0.7 μM Ttc (red trace; **A**_2_). *I*_*p*_ and *I*_*ss*_ values are indicated by arrows in the *I*_*ACh*_s elicited solely by ACh (*I*_*p*_*Ctr*_ and *I*_*ss*_*Ctr*_), or together with Ttc (*I*_*p*_*Ttc*_ and *I*_*ss*_*Ttc*_). Note that *I*_*p*_*Ttc*_ was reached earlier than *I*_*p*_*Ctr*_. **(B)** Relationship between changes in *I*_*ACh*_ desensitization (see Equation 2) and extent of *I*_*ss*_ inhibition evoked by different concentrations of Ttc (0.01–2 μM). The black discontinuous line is a reference indicating no change in desensitization and the blue line is the best linear fit to values falling below the reference line (0.1–2 μM Ttc). Each point represents the average obtained from 7 to 19 oocytes (*N* = 2–9), except for 0.5 μM Ttc, in which *n* = 3 and *N* = 1. Asterisks indicate significant differences from control desensitization (*p* < 0.05, one-sample *t*-test).

Further evidence indicating that Ttc promotes faster nAChR desensitization arise from computation of the ratios of *I*_*ss*_ vs. *I*_*p*_ amplitudes, when co-applying 10 μM ACh with different concentrations of Ttc, as proposed by Sobolevsky et al. (1999) (see Equation 2 in section Materials and Methods and Figure 8). At low concentrations of Ttc (lower than 0.5 μM), the quotient of the *I*_*ss*_ to *I*_*p*_ ratio in the presence of Ttc (*I*_*ss*_*Ttc*_*/I*_*p*_*Ttc*_) over the *I*_*ss*_ to *I*_*p*_ ratio in the presence of ACh alone (*I*_*ss*_*Ctr*_*/I*_*p*_*Ctr*_) was close to 1. However, at higher Ttc concentrations (0.5 μM or above), this quotient was significantly smaller than 1 (*p* < 0.05; one-sample *t*-test), and interestingly, was reduced in a dose-dependent manner, as the extent of *I*_*ss*_ inhibition by Ttc increased (Figure 8B). Therefore, the plot in Figure 8B illustrates that low Ttc concentrations elicit nAChR blockade without affecting *I*_*ACh*_ decay, whereas Ttc concentrations over 0.5 μM evoke both (i) *I*_*ACh*_ reduction by nAChR blockade; and (ii) acceleration of *I*_*ACh*_ decay by enhancement of nAChR desensitization.

**Figure 9:**
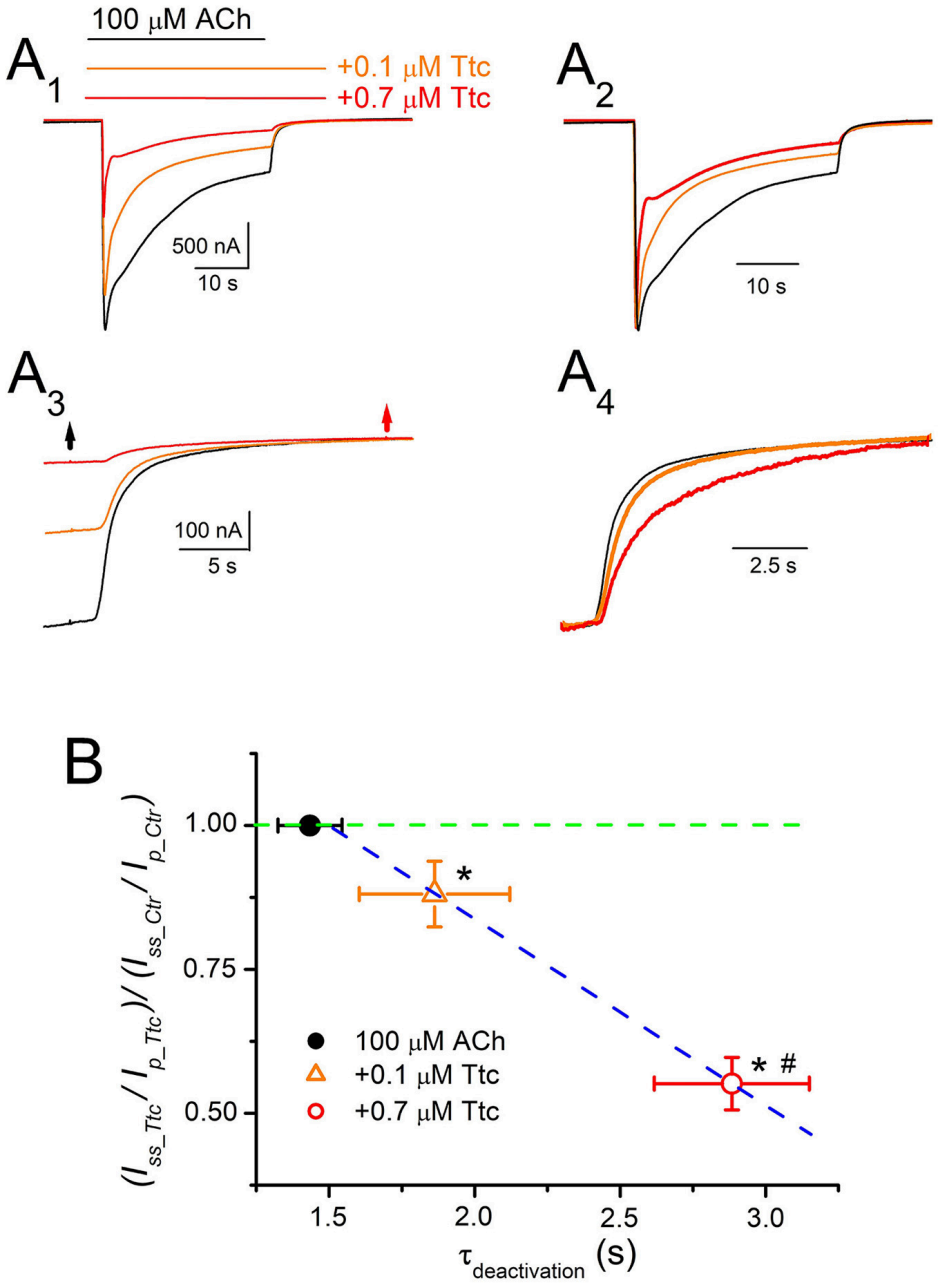
Deactivation kinetics of currents elicited by ACh (*I*_*ACh*_) are dependent on the concentration of tetracaine (Ttc). **(A)** Representative *I*_*ACh*_s elicited by 100 μM ACh, either alone (black recording), or together with 0.1 μM (orange) or 0.7 μM (red) Ttc **(A**_1_**)**. Superfusion of Ttc lasted 12 s after ACh washout. These recordings were normalized to the same *I*_*p*_ **(A**_2_**)** to show changes in desensitization more effectively. **(A**_3_**)** Deactivation of *I*_*ACh*_s shown in **(A**_1_**)**. The black arrow indicates ACh washout and the red arrow indicates Ttc removal. **(A**_4_**)** *I*_*ACh*_ deactivations shown in **(A**_3_**)** were scaled to the same amplitude to better compare their time course. **(B)** Relationship between desensitization changes (Equation 2) and the apparent deactivation time constant (τ_apparent−deactivation_) elicited by 0.1 μM (orange triangle) and 0.7 μM (red circle) Ttc. The black filled symbol corresponds to the τ_apparent−deactivation_ of *I*_*ACh*_s elicited by ACh alone, which is rate limited by the solution exchange kinetics. The green discontinuous line indicates the control desensitization ratio. Note the higher desensitization rate elicited by Ttc (values lower than 1 in the ordinate), and the slower deactivation rate (higher τ_apparent−deactivation_ values in the abscissa) following a linear relationship (blue discontinuous line). Each point represents the average of 12–25 oocytes from eight donors. Asterisks indicate significant differences in desensitization and deactivation (*p* < 0.05, *t*-test), with respect to control values and the pound means differences in both parameters depending on the Ttc concentration used (*p* < 0.05, *t*-test).

On the other hand, the kinetics of *I*_*ACh*_ tails (deactivation) differed when ACh was withdrawn, but Ttc remained in the ANR. In these experiments, 100 μM ACh, which evokes considerable nAChR desensitization, was co-applied with either 0.1 or 0.7 μM Ttc for 32 s, and ACh was then washed out, while keeping the cell superfused with Ttc for an additional 12 s. As previously shown, the effects of Ttc on *I*_*ACh*_ are concentration-dependent. Thus, 0.1 μM Ttc elicited both a small *I*_*ACh*_ reduction, and a slight, but significant, enhancement of *I*_*ACh*_ decay, mainly of the fast desensitization component (Figures 9A_1_,A_2_). Thus, the ratio of *I*_*ACh*_ decay time constant values obtained in the presence of 0.1 μM Ttc vs. ACh alone was significantly smaller than 1 (0.69 ± 0.07; *n* = 12, *N* = 6; *p* < 0.05, one-sample *t*-test). Notably, co-application of 10 μM ACh together with 0.1 μM Ttc did not modify *I*_*ACh*_ decay (Figures 7A_2_,B), whereas the same concentration of Ttc co-applied with 100 μM ACh significantly accelerated *I*_*ACh*_ decay (Figure 9A_2_). When 100 μM ACh was co-applied with 0.7 μM Ttc, nAChR blockade was increased and *I*_*ACh*_ decay was accelerated (Figures 9A_1_,A_2_). The ratio of *I*_*ACh*_ decay time constant values obtained in the presence of 0.7 μM Ttc and ACh alone was 0.23 ± 0.08 (*n* = 13, *N* = 8). This indicates that at this Ttc dose, the *I*_*ACh*_ decayed significantly faster than in the presence of 0.1 μM Ttc (*p* < 0.05, *t*-test). Moreover, *I*_*ACh*_ deactivation after ACh withdrawal, followed a single exponential time course, the kinetics of which was affected by keeping Ttc in the ANR (Figures 9A_3_,A_4_). Deactivation of control *I*_*ACh*_s, elicited by ACh alone, followed an exponential function with a time course that was limited by the solution exchange kinetics (time constant of 1.4 ± 0.1 s, *n* = 25; see Figure 5); thus, we would refer to these values as apparent deactivation time constants (τ_apparent−deactivation_). The presence of Ttc decelerated *I*_*ACh*_ deactivation in a dose-dependent manner (τ_apparent−deactivation_ of 1.9 ± 0.3 and 2.9 ± 0.3 s for *I*_*ACh*_s in the presence of 0.1 and 0.7 μM Ttc, respectively; same cells as above; Figures 9A_3_,A_4_,B), as ACh washout kinetics remained constant. Deceleration of *I*_*ACh*_ deactivation would be expected if Ttc enhances nAChR desensitization, as has been previously reported for GABA_A_Rs (Jones and Westbrook, 1995). Accordingly, a linear correlation exists between the extent of desensitization (see Equation 2 in section Materials and Methods) and the apparent deactivation time constant values in the presence or absence of Ttc (Figure 9B).

### Virtual docking assays

The interactions between Ttc and the nAChR were explored by using the full structure of *Torpedo* nAChRs as a template in both the open and closed conformations (Alberola-Die et al., 2016a,b). For each conformation we carried out 800 runs to assess Ttc-nAChR interactions. We found 279 clusters of interaction sites differing by less than 5 Å of the root-mean-square-deviation for the nAChR in the open state and 257 in the closed state. As shown in Figure S3 (open state) and Figure S4 (closed state), these clusters were located at the ECD (87 and 89% for open and closed states, respectively) and at the TMD (13 and 11% for open and closed states, respectively). No clusters were found at the intracellular domain (ICD). Most nAChR-Ttc interactions at the ECD involved two subunits (53 and 57% for open and closed states, respectively), mainly α_1_-γ, α_2_-δ, close to the orthosteric binding site, and α_1_-β for both the open and closed states (Figures S3Cc_1_, S4Cc_1_). In contrast, TMD clusters were involved in simultaneous binding to residues of 3–5 subunits deep within the channel pore (Figures S3Cc_2_, S4Cc_2_). In addition, there were TMD clusters located close to the ECD-TMD interface, at intra- or inter-subunit crevices, comprising 1 or 2 subunits, respectively (Figures S3Cc_3_,c_4_, S4Cc_3_,c_4_). Notably, Ttc was bound to roughly the same nAChR residues within the channel pore in both open and closed states (see Table 1). Conversely, less overlapping was observed with respect to Ttc binding to residues at the ECD and inter- and intra-subunit crevices of the TMD. Thus, only 22 out of 47 (46.8%) residues that were bound to Ttc at the ECD in the closed state were also bound to this LA in the open state (Table 1). A similar percentage of coincidence was found when considering intra- and inter-subunit residues of the TMD, specifically 16 out of 26 (61.5%) and 14 out of 33 (42.4%), respectively (Table 1).

**Table 1:** Nicotinic acetylcholine receptor (nAChR) residues interacting with tetracaine (Ttc) in open and resting (closed) states.

**Receptor state**	**Domain (location)**	**Interfaces**	**Subunits**	**Residues**
**Open**	**EC**	α-γ	α_γ_	W149, T150, Y151, D152, Y190, P197, Y198
			γ	R78, Y116, L118, P120
		α-δ	α_δ_	W149, T150, Y151, D152, P197, Y198
			δ	S40, N55, W57
		α-β	α_γ_	T106, K107, L108, L109, M117, W118, T119, P120
			β	Y149, T150, Y151, D152
	**TM** (M2)		α_γ_/α_δ_	S248, L251, S252, V255, F256, E262, L263
			β	S254, L257, A258, V261, F262
			δ	C262, L265, A266, V269, F270
			γ	N224, S256, L259, A260, I263, F264, L267, Q270, K271, E274
	**TM** (intersubunit)	β-δ	β	F219, Y220, V222, Y223
			δ	L287, I288, G289, L292, M296
		α-γ	α_δ_	F214, N217, V218, I220, P221, L224
			γ	T262, L265, F266, A269, P273
	**TM** (intrasubunit)		α_γ_/α_δ_	I264, L273, Y277, M278, F280, T281, F284
			β	K269, V270, S274, P278, I279, I280, I281, Y283
			δ	K224, Y228, F232, I233, L278, L287, Y291, F294, I295
			γ	L219, I222, I225, I226, Y285, F288, V289, T468
**Resting**	**EC**	α-γ	α_γ_	V91, L92, Y93, A96, I148, **W149, T150, D152, Y198**
			γ	W54, **R78**, L108, **Y116, L118**
		α-δ	α_δ_	V91, L92, Y93, N95, A96, I148, **W149, Y198**
			δ	**S40**, N41, **N55, W57**, V104, P123, I125
		α-β	α_γ_	R55, **T106, K107, L108, L109, W118, T119, P120**, P121
			β	V91, L92, N96, G98, S99, F100, **Y149, T150, Y151**
	**TM** (M2)		α_γ_/α_δ_	**S248, L251, S252, V255, F256**, **E262**
			β	**S254, L257, A258, V261, F262**
			δ	**C262, L265, A266, V269, F270**
			γ	**N224**, **S256, L259, A260, I263, F264**, **K271**
	**TM** (intersubunit)	β-δ	β	P217, L218, **F219, Y220**, I221, **V222, Y223**
			δ	L278, P279, A282, L283, V285, P286, **L287, L292**
		α-γ	α_δ_	**N217, I220, P221, L224**, F225, L228, L253, F256, I260
			γ	**T262, L265, F266**, I268, **A269**, M290, S293, L294, V297
	**TM** (intrasubunit)		α_γ_/α_δ_	**L273, Y277, M278, F280, T281, F284**
			β	**V270, S274**, V277, **P278, I279, I280, I281, Y283**
			δ	**Y291, F294**, L298, G301, V302, N305
			γ	I268, **F288, V289**, V292, S293, I296

*Main nAChR residues at extracellular (EC) or transmembrane (TM) domains, where Ttc binds when the receptor is in the open or the resting state. Red labeled residues are located at a shallow depth within the channel pore and seem to be involved in enhancement of the desensitizing effects of Ttc. Coincident interacting residues in both nAChR configurations are indicated in bold font in the resting state*.

**Figure 10:**
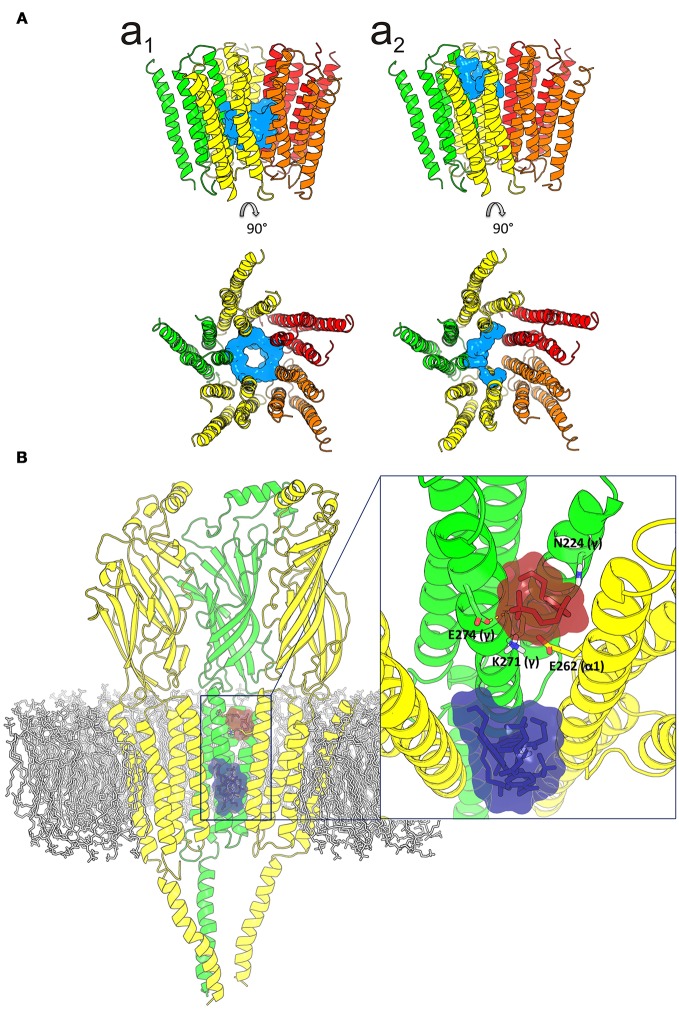
Idealization of two putative tetracaine (Ttc) binding sites within the channel pore. **(A)** Lateral (upper) and top (lower) views of the transmembrane domain (TMD), in the open nicotinic acetylcholine receptor (nAChR), showing Ttc (highlighted in cyan) bound at the higher affinity (at the middle of the pore; **a**_1_) and lower affinity (closer to the extracellular side; **a**_2_) sites. **(B)** Lateral view of the three nAChR domains (membrane bilayer in gray). The two Ttc binding loci within the channel pore are highlighted by a square. A zoomed in image of this frame, from the synaptic cleft, is shown on the right. Ttc molecules (in purple) were used to block the high-affinity site within the pore, to reveal the Ttc low-affinity binding site (Ttc interacting molecules shown in brown), which includes E262(α), N224(γ), K271(γ), and E274(γ) as key interacting residues.

Given the strong effect of Ttc on *I*_*ACh*_ decay elicited by the enhancement of nAChR desensitization, and the presence of this effect only at negative potentials (see Figure 4), it is logical to consider that Ttc increases desensitization through its binding within the channel pore. To assess this hypothesis, we performed additional docking assays focused just at the channel pore when it was in the open conformation. Further, 150 runs were performed to assess Ttc-nAChR channel pore interactions and docking developed at the same residues located at the middle of the channel pore, in a similar manner to those reported above, using the whole nAChR structure (Figure S3Cc_2_, and Figure 10Aa_1_). In an attempt to explore further Ttc-nAChR interactions in the channel, we blocked Ttc binding at the residues indicated above. Additional runs carried out under the specified conditions showed that Ttc also binds, although with a lower affinity, to residues of α_1_, α_2_ (E262 and L263), and γ subunits (N224, L267, Q270, K271, and E274), located at the channel pore, close to the extracellular side (Figures 10Aa_2_,B; Table 1). Interestingly, when this approach was repeated using the nAChR in the closed conformation, we found that Ttc was bound to coincident sites within the channel pore, despite the fact that the sole pre-application of Ttc did not affect the rate of *I*_*ACh*_ decay (Figure 4A_2_).

Table 2 reflects the theoretical binding energy and K_d_ values (Equation 4) of Ttc docking solutions at the three main nAChR binding sites both in the resting and open states: *site-1*, the ECD; *site-2*, the outer mouth of the pore; and *site-3*, located deeper within the channel pore (see image of Table 2). In the resting state, the binding energy of site 2, which corresponds to the low-affinity site, was lower than those of either sites 1 (ECD) or 3 (the high-affinity site within the pore). Similarly, in the open state, the Ttc binding energy of site 2 was significantly lower than those observed for sites 1 and 3 (*p* < 0.05, *t*-test). In contrast, the ECD sites presented similar K_d_ values, both in the open and resting states, and they were comparable to those of site 3 in the closed state. Additional binding energy data with details of interfaces and locations are depicted in Table S1.

**Table 2:** Binding energies and dissociation constants (K_d_) of tetracaine (Ttc) bound to closed and open muscle-type nicotinic acetylcholine receptors (nAChRs).

**Receptor state**	**Binding site location**	**Mean binding energy (kcal/mol)**	**(*n*)**	**K_d_ (M)**	
Resting	1	4.79 ± 0.54	133	3.08 × 10^−4^	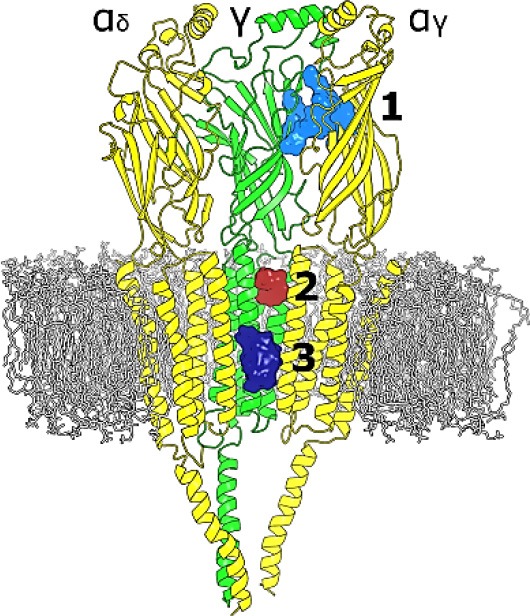
	2	3.67 ± 0.67*	12	2.05 × 10^−3^	
	3	4.75 ± 0.19	66	3.31 × 10^−4^	
Open	1	4.53 ± 0.66	107	4.74 × 10^−4^	
	2	3.48 ± 0.36*	33	2.79 × 10^−3^	
	3	3.94 ± 0.51^#^	58	1.28 × 10^−3^	

*The main Ttc binding sites are denoted as (1) at the extracellular domain (ECD); (2) at the low-affinity site within the pore; and (3) at the high-affinity site within the pore. See right-hand figure for their location within the nAChR. Different binding energies of the same locations were averaged to get a single energy value, which is indicated together with its standard deviation. The K_d_s were calculated using Equation 4 (see section Materials and Methods). Asterisks indicate significant differences between Ttc binding energies of site 2 and those of either sites 1 or 3, in both the open and closed conformations (p < 0.05, t-test); the pound sign denotes significant differences between sites 1 and 3 in the open state; n is the number of docking solutions that were averaged*.

When the present manuscript was under review, Newcombe et al. (2018) published a refined structural model of the nicotinic α_7_ subunit. This model corrects a previously identified error in the TMD alignment of *Torpedo* subunits, which mainly involves a shift of one helix turn at the base of the M1-M2 helices. As a refined model for the different subunits forming the muscle-type nAChR is not available, we used the structure of homomeric α_7_ nAChRs, in both the open and closed states, to assess the relevance of the M1-M2 loop and nearby M2 residues in Ttc binding. After conducting 800 docking runs in each conformation, we found no Ttc interactions on the M1-M2 loop or nearby residues of the M2 helix (see Figure S5). Therefore, the M1-M2 loop and nearby residues do not appear to be relevant targets of Ttc, at least in the homomeric nAChR. In addition, the inaccuracies of the original *Torpedo* structural model do not seem to substantially affect the docking results presented above.

## Discussion

This work confirms that Ttc is a powerful blocker of muscle-type nAChRs and deepens our understanding of the modulatory mechanisms of LAs associated with nAChR function. Along with other LAs that possess tertiary amine groups, such as lidocaine, Ttc shares some similar effects on nAChRs. However, there are also significant differences among their effects. For instance, the potency of Ttc as a nAChR blocker (*IC*_*50*_ of 0.3 and 0.7 μM, for the *I*_*ss*_ and *I*_*p*_, respectively) is comparable to that of d-tubocurarine, and markedly higher than those of lidocaine (11–73 μM) (Gentry and Lukas, 2001; Alberola-Die et al., 2011) or procaine (25–230 μM) (Adams, 1977; Koblin and Lester, 1979; Gentry and Lukas, 2001; Wang et al., 2010). Notably, the Ttc *IC*_*50*_ here reported is roughly one order of magnitude smaller than the value found for muscle-type nAChRs expressed in a human cell line (TE671/RD; 13 μM) (Gentry and Lukas, 2001) and roughly one hundredth the *IC*_*50*_ reported for mouse cells (BC3H-1; 38 μM) (Eterović et al., 1993). Nevertheless, our Ttc *IC*_*50*_ was similar to the value stated for nAChRs from the electric organ of *Torpedo californica* expressed in *Xenopus* oocytes (1 μM) (Eterović et al., 1993), but smaller than that obtained for nAChRs from *Electrophorus electricus* electroplaques (25 μM) (Koblin and Lester, 1979). The large differences in the blocking potency of Ttc among nAChRs from the electric organ and muscle-type nAChRs expressed in diverse cells lines could be due to the different subunit conformations of these receptors; specifically, the synaptic-type (ε-like subunit) composition of the former and the extrasynaptic-type (γ-like subunit) composition of the latter. Nevertheless, whether or not muscle-type nAChRs from fetal (or denervated muscle) and adult muscles have the same sensitivity to Ttc remains to be assessed.

The main effect of Ttc on nAChRs at low concentrations, below its *IC*_*50*_, was a voltage-dependent blockade (Figures 2A_1_,B_1_), indicating that Ttc enters the channel pore. This blockade resembles that elicited by other LAs and compounds with tertiary amine groups, such as lidocaine (Alberola-Die et al., 2011), procaine (Adams, 1977), and DEA (Alberola-Die et al., 2016a). The kinetics of the voltage-dependent blockade was assessed by stepping back the cell membrane potential from +40 mV (which ejects Ttc from its binding site within the channel) to −60 mV, while the cell was superfused with 10 μM ACh and Ttc. At 0.1 μM, Ttc most likely binds only to the high-affinity site (site 3) within the channel, and the elicited voltage-dependent blockade could be fitted by a single exponential function. Notably, at 0.7 μM Ttc (a concentration at which Ttc might bind to sites 2 and 3), the blocking kinetics also followed a single exponential function, but with a faster time constant, as would be expected with an increase in the number of blocking molecules. Therefore, these results suggest that most of the voltage-dependent blockade by Ttc is due to its binding to site 3. In accordance with these findings, the slope of the voltage-dependent blockade at negative potentials remains essentially unaltered at different concentrations of Ttc (see Figure S2). It should be noted that the voltage-dependent blockade of nAChRs by charged molecules is intermittent, with very fast kinetics, causing the characteristic current “flickering” (Neher and Steinbach, 1978). In contrast, it is likely that the effect on desensitization is a more long-lasting phenomenon, as it involves conformational changes. Thus, it is possible that Ttc binding to site 2 mainly promotes nAChR desensitization, leading to non-conducting desensitized nAChR, instead of a plain open-channel blockade, which is more easily reversible. At high concentrations (*IC*_*50*_ or above), Ttc also elicited a voltage-independent blockade of nAChRs (Figures 2A_2_,B_2_). This additional nAChR blockade is most likely due to the action of Ttc on nAChR residues located outside the channel pore, as it was also present at positive potentials, which should have removed most of the positively charged Ttc from the pore. Nevertheless, according to the Woodhull model (Woodhull, 1973), some *I*_*ACh*_ inhibition could be observed at positive potentials when using relatively high blocker concentrations, if the blocker binds at a shallow site within the channel pore, as Ttc does (see the mild slope of the voltage-dependent blockade of *I*_*ACh*_ in Figure S2). Even so, the charged cholinesterase inhibitor BW284c51, which elicits open-channel blockade of *Torpedo* nAChRs through its binding to a shallow site within the channel (delta of 0.1; i.e., very close to the extracellular side) (Olivera-Bravo et al., 2007) does not inhibit *I*_*ACh*_ at positive potentials, when tested at its *IC*_*50*_ (Olivera-Bravo et al., 2005, 2007). Moreover, the hypothesis of a single Ttc binding site cannot explain several key experimental results reported in the present study, such as: (i) the marked differences between Ttc unbinding kinetics when Ttc is just pre-applied (either at −60 or +40 mV; Figure 4A_2_,A_5_) and when it is applied during the *I*_*ACh*_ (Figure 5); (ii) the higher nAChR blockade evoked by 0.7 μM Ttc when it is pre-applied and then co-applied with ACh, as compared to the effect of just Ttc and ACh co-application (Figures 4B_1_,B_2_); (iii) the changes *in I*_*ACh*_ decay, found solely when ACh was co-applied with Ttc at concentrations close to, or above, the *IC*_*50*_ (Figure 7B); (iv) the slower *I*_*ACh*_ deactivation in the presence of Ttc, and its correlation with the acceleration of *I*_*ACh*_ decay (Figure 9B), which strongly suggests that both are due to the enhancement of nAChR desensitization; (v) the lack of acceleration in *I*_*ACh*_ decay by Ttc when holding the membrane potential at positive potentials (compare recordings Figure 4A_1_ and Figure 4A_4_); and (vi) the different nAChR-Ttc interaction sites found in our docking assays, associated with both the ECD and TMD (see inset of Table 2 and Figures S3, S4). All these experimental data provide strong evidence (although not irrefutable proof) that the effects of Ttc on *I*_*ACh*_ are mediated by its binding to multiple sites in the nAChR.

As indicated above, Ttc increased its inhibitory effect when pre-applied to the cell, before its co-application with ACh (Figure 3B,C), suggesting a resting-channel blockade. Indeed, Ttc binding to closed nAChRs has been previously reported, either by measuring the inhibition of labeled perhydrohistrionicotoxin binding to nAChR-enriched membrane fragments (Blanchard et al., 1979; Middleton et al., 1999) or by photolabeling nAChR-rich membranes with radioactive Ttc (Gallagher and Cohen, 1999; Middleton et al., 1999). These authors found a Ttc binding affinity roughly 30-fold higher in the resting state than in the desensitized state (*IC*_*50*_*s* of ≈ 1 μM vs. 30 μM, respectively; Blanchard et al., 1979; Middleton et al., 1999), and reported that Ttc binds within the channel pore while the nAChR is in the closed state (Gallagher and Cohen, 1999). Accordingly, our docking assays on nAChRs showed that Ttc binds within the channel pore, both in the open and closed conformations (site 3 of Table 2; Figures S3A,Cc_2_, S4A,Cc_2_, respectively), and become involved with the same residues in both states (see Table 1). Notably, the M2 residues interacting with Ttc at the middle of the channel pore are roughly the same as those reported for Ttc interactions with resting nAChRs (Gallagher and Cohen, 1999). Although our *in silico* results do not show that M2 residues had a higher Ttc binding affinity when the nAChR was in the open state, the selective open-channel blockade elicited by low concentrations of Ttc indicate that these residues should have had the highest affinity for Ttc. Nevertheless, the docking data revealed that the Ttc binding energies of site 3 were significantly higher than those of site 2 (located at a shallower depth in the TMD; see Table 2) and roughly similar to those of site 1 (binding sites at the ECD). Our virtual docking assays on the resting nAChR showed that Ttc interacts mostly with residues located at the ECD (Figure S4). Furthermore, the functional results suggest that Ttc binds to different (independent) sites, whether the nAChR is closed or open (Figure 4), and this binding is dependent on the concentration of Ttc administered. In this regard, it should be pointed out that the concentration of Ttc used for the photolabeling experiments was 5 μM (Gallagher and Cohen, 1999), which is almost one order of magnitude higher than our *IC*_*50*_ value, and roughly 50-fold the concentration of Ttc that elicits selective voltage-dependent blockade of nAChRs (Figures 2A_1_,B_1_).

As would be expected from the above-mentioned open- and closed-channel blockade, the pharmacological profile of nAChR inhibition by Ttc followed a non-competitive pattern (Figure 3B). Therefore, the extent of *I*_*ACh*_ inhibition was independent of agonist concentration (Figure 3C), although it was affected by the timing of Ttc application (direct co-application ACh and Ttc *vs*. pre-application of Ttc, followed by its co-application with ACh). Interestingly, when 0.7 μM Ttc was pre-applied alone, the *I*_*ACh*_ inhibition elicited either at −60 or +40 mV, showed almost no recovery during the following 32 s pulse of ACh (Figures 4A_2_,A_5_,B_1_,B_2_). We could speculate that Ttc partition in the membrane would account for this slow *I*_*ACh*_ recovery. Indeed, protonated Ttc, similar to other molecules with charged ammonium groups, could interact with negatively-charged phosphate groups of membrane phospholipids through long-range coulombic interactions (Pérez-Isidoro et al., 2014). However, membrane adsorption of Ttc at the concentrations used in the present study (below 1 μM) does not seem to sufficiently explain the delayed and long-lasting nAChR blockade found with just pre-application of Ttc. Instead, we think that the sustained nAChR blockade when Ttc was solely pre-applied would be due to Ttc binding outside the channel pore in resting nAChRs (closed-channel blockade), as it was found at both negative and positive potentials (Figures 4A_2_,A_5_). Accordingly, Figure 5 shows that Ttc “off” rate kinetics of *I*_*ACh*_ (roughly 3 s; Figure 5C) is only moderately slower than its corresponding “on” rate (time constant values circa 1.5 s), but much faster than the *I*_*ACh*_ recovery observed after Ttc pre-application alone. Furthermore, quaternary ammonium molecules, such as BW284c51 or edrophonium, show similar washout kinetics (in the range of a few seconds) (Olivera-Bravo et al., 2007), even if they are superfused at very different concentrations (0.5 μM and 10 μM for BW284c51 and edrophonium, respectively). Furthermore, Leng et al. (2013) reported the Ttc inhibition of ASIC3 channels by repeating, within the same cell, pH pulses with increasing concentrations of Ttc, up to 30 mM. Despite the high Ttc doses used in that study (over four orders of magnitude above those used in the present study to block nAChRs), no additive effects were apparent when pulses were repeated at 90 s intervals. All of these experimental findings contradict the possibility that the membrane acts as a large reservoir for lipid-partitioned Ttc molecules, which would slowly release Ttc after being washed out from the solution, and thus, sustain nAChR inhibition over time.

When Ttc dose-nAChR inhibition curves were plotted for both *I*_*p*_ and *I*_*ss*_ values (Figure 1C), they showed that Ttc inhibition was rather similar for both components up to 0.1 μM Ttc. However, at higher concentrations of Ttc, there was increased inhibition at the *I*_*ss*_. This increase in *I*_*ACh*_ blockade at its steady state is directly related to the enhancement of *I*_*ACh*_ decay, which requires the action of Ttc within the channel pore. Thus, it was not observed when the cell membrane was maintained at positive potentials, which eject the positively charged Ttc from the channel pore (compare recordings of Figures 4A_1_,A_4_). The acceleration of *I*_*ACh*_ decay by Ttc might be mediated by either a slow-pace blockade of nAChRs, enhancement of desensitization, or a combination of both factors. However, we have assembled several experimental findings that support the hypothesis that the main reason is an increase in the rate of nAChR desensitization. First, the same Ttc concentration (0.7 μM) accelerated *I*_*ACh*_ decay more sharply when it was co-applied with 100 μM ACh (τ_Ttc_ = 0.6 s) than with 10 μM ACh (τ_Ttc_ = 1.0 s) (Figure 6). Second, as already mentioned, Ttc at its *IC*_*50*_ blocked nAChRs at both negative and positive potentials, but *I*_*ACh*_ decay was only enhanced when the cell was maintained at negative potentials (Figures 4A_1_,A_4_). Third, the Ttc blocking kinetics was faster than the acceleration of *I*_*ACh*_ decay induced by Ttc. Thus, the time course of the voltage-dependent blockade of nAChR by 0.7 μM Ttc was faster (Figure 2C) than the *I*_*ACh*_ decay evoked by the same concentration of Ttc, even when co-applied with a high concentration of ACh (100 μM; τ_Ttc_ = 0.6 s, Figure 6). Fourth, if Ttc would accelerate *I*_*ACh*_ decay because of a slow-pace blockade of nAChRs, it should be detected at all concentrations of Ttc that induce *I*_*ACh*_ inhibition. However, 0.1 μM Ttc, which inhibits roughly 25% *I*_*ACh*_ (Figures 1, 7), does not modify *I*_*ACh*_ decay (Figure 7). Fifth, at Ttc concentrations above 0.5 μM, the ratio *I*_*ss*_/*I*_*p*_ vs. its corresponding control value (in the presence of 10 μM ACh alone) is significantly smaller than 1 (Figure 8B), indicating an enhancement of nAChR desensitization (Sobolevsky et al., 1999). In contrast, below 0.5 μM Ttc, this quotient is close to 1 (Figure 8B). Sixth, Ttc decelerated *I*_*ACh*_ deactivation (Figure 9). The pronounced deceleration of *I*_*ACh*_ deactivation elicited by Ttc, when applied at its *IC*_*50*_, also indicates an enhancement of nAChR desensitization, because of the higher affinity of the desensitized nAChR to the agonist, as previously suggested for GABA_A_Rs (Jones and Westbrook, 1995). Nevertheless, the solution exchange kinetics of our experimental model limits the temporal resolution to roughly 1.4 s (Figure 5). Therefore, to assess the kinetics of the voltage-dependent *I*_*ACh*_ blockade, the cell membrane potential was jumped from positive to negative voltages in the presence of Ttc, which facilitated the measurement of this kinetics independently of the solution exchange rate (Figure 2). However, as *I*_*ACh*_ deactivation kinetics is affected by the solution exchange rate, we referred to the observed values as apparent deactivation time constants, to indicate this limitation.

Altogether, the aforementioned results indicate that Ttc indeed enhances nAChR desensitization. Furthermore, both our functional and virtual docking results support the notion that Ttc accelerates nAChR desensitization by binding to M2 residues located at the interphase between the ECD and TMD (site 2 of Table 2), a region that is relevant to the determination of both the open-channel lifetime and rate of desensitization of Cys-loop receptors (Bouzat et al., 2008). Thus, our functional studies indicate that Ttc requires binding within the channel to boost desensitization, as *I*_*ACh*_ decay is not affected by Ttc at positive membrane potentials, which eject Ttc from the channel lumen. Consistent with these findings, our docking assays indicated that Ttc binds to residues of the α and γ subunits located at a very shallow depth within the channel pore (Figures 10Aa_2_,B and Tables 1, 2), both in the open and closed states. Interestingly, one of these residues is αE262, which is located at the extracellular end of the channel pore. This residue is highly conserved among different nAChRs subtypes and has been involved in the desensitization/resensitization of *Torpedo* nAChRs (Forman et al., 2007). Indeed, αE262 mutants have the fast component of nAChR desensitization altered, and photomodification of αE262 with 3-azioctanol stabilizes the desensitized state (Forman et al., 2007). In addition, crystal violet, a nAChR antagonist, reportedly enhances the desensitization of resting receptors, likely by binding to αE262, and stabilizes the desensitized state (Arias et al., 2006). Notably, Ttc inhibits crystal violet binding to resting AChRs (Arias et al., 2006), suggesting that both molecules interact at the same, or nearby sites. However, in our hands, Ttc did not promote changes in desensitization when acting on resting nAChR, as the *I*_*ACh*_ decay was not accelerated by the sole pre-application of Ttc, i.e., when Ttc acted on resting receptors only. Moreover, if Ttc had desensitized resting nAChRs, a slow increase of the *I*_*ACh*_ would be expected during the subsequent ACh application, because of the slow recovery of nAChR from desensitization; however, no changes in *I*_*ACh*_ decay were observed with this protocol (Figure 4A_2_). In contrast, Ttc binding to site 2 enhances nAChR desensitization when the channel is in the open conformation, as evidenced by the accelerated *I*_*ACh*_ decay observed when Ttc and ACh were co-applied, either directly, or following a 12 s pre-application of Ttc (Figures 4A_1_,A_3_). Interestingly, this superficial binding site in the channel pore differs from another, more deeply located within the channel (site 3), to which Ttc binds with higher affinity, thereby eliciting open-channel blockade (steric blockade; Figure 10). Nevertheless, our docking assays used the structural models of *Torpedo* nAChR derived from cryo-eletron microscopy as a template, which bears a rather low resolution, particularly in the open-channel model (6.2 Å) (Unwin, 1995). Moreover, these templates contain an error in the TMD alignment that is mainly associated with a shift of one helix turn at the base of the M1-M2 helices. Nevertheless, this TMD region does not appear to be a target of Ttc, neither when the original *Torpedo* templates were used, nor when the homomeric α_7_ nAChR refined structure was used (Newcombe et al., 2018). This suggests that the inaccuracies of the original *Torpedo* structural model do not substantially affect the docking results presented. Thus, our virtual docking assays provide a coherent explanation of our experimental observations, in terms of the involvement of different sets of Ttc binding sites that account for its complex modulating actions on nAChRs.

In conclusion, our present results indicate that Ttc, a molecule that is widely used in clinical practice for both topical and spinal administration, should no longer be considered only as a non-competitive blocker of nAChRs that selectively act on the resting (closed) state (Middleton et al., 1999). Here, we provide strong functional evidence indicating that Ttc is a very powerful blocker of muscle-type nAChRs, with an *IC*_*50*_ in the submicromolar range, which acts on both the closed and open states of nAChRs. Furthermore, Ttc greatly enhances nAChR desensitization, most likely by binding to the most superficial region of the pore when the channel is in the open conformation. It is worth noting that as around 100 μM Ttc is required to inhibit 80% of voltage-dependent Na^+^ channels (Wang et al., 1996), the high potency of Ttc inhibiting muscle-type nAChRs, and perhaps other neuronal subtypes of nAChRs, might explain some of its serious side effects, despite the fact that it is rapidly hydrolyzed by plasma esterases (Moriya, 2005). Nevertheless, Ttc concentrations in plasma of up to 0.7 μM have been reported in humans after its topical application on the skin (2 g, 5% w/w), without any remarkable side effects (Mazumdar et al., 1991). Although roughly all Ttc molecules in physiological solutions are protonated, in contrast to other LAs with amine groups, Ttc binds to different nAChR loci, which accounts for the heterogeneity of its functional effects on nAChRs. These results contribute to a better understanding of the complex modulation of muscle-type nAChRs by Ttc, and they provide new insights about the key nAChR loci involved in both allosteric and steric modulation.

## Author contributions

All authors listed have made a substantial, direct and intellectual contribution to the work, and approved it for publication.

### Conflict of interest statement

The authors declare that the research was conducted in the absence of any commercial or financial relationships that could be construed as a potential conflict of interest.

## Abbreviations

ACh: acetylcholine
ANR: normal Ringer solution with atropine
DEA: diethylamine
DMA, 2: 6-dimethylaniline
EC: extracellular
*I*_*ACh*_: ACh-elicited current
IC: intracellular
*I*_*p*_: *I*_*ACh*_ amplitude at the peak
*I*_*ss*_: *I*_*ACh*_ amplitude at the steady-state
LA: local anesthetic
MS-222: ethyl 3-aminobenzoate methanesulfonate
*n*: number of oocytes
*N*: number of oocyte-donor frogs
nAChR: nicotinic acetylcholine receptor
NR: normal Ringer solution
TM: transmembrane spanning-segment
Ttc: tetracaine.
