# Supplementary material for: Mechanisms Underlying the Strong Inhibition of Muscle-Type Nicotinic Receptors by Tetracaine
Source: Front Mol Neurosci. 2018 Aug 8;11:193. doi: 10.3389/fnmol.2018.00193 (PMC6092513; doi:10.3389/fnmol.2018.00193)
Supplement: Supplementary file 1 [file Image_1.pdf]

Supplementary Material

Mechanisms underlying the strong inhibition of muscle-type nicotinic receptors by tetracaine

Raúl Cobo[1], Magdalena Nikolaeva[2], Armando Alberola-Die[1], Gregorio Fernández-Ballester[2], José Manuel González-Ros[2], Isabel Ivorra[1] and Andrés Morales[1,*]

[1]División de Fisiología, Departamento de Fisiología, Genética y Microbiología, Universidad de Alicante, Apdo. 99, E-03080 Alicante, Spain.

[2]Instituto de Biología Molecular y Celular, Universidad Miguel Hernández, Elche, E-03202, Alicante, Spain

*** Correspondence:**
Andrés Morales
andres.morales@ua.es
Phone: 34-965903949

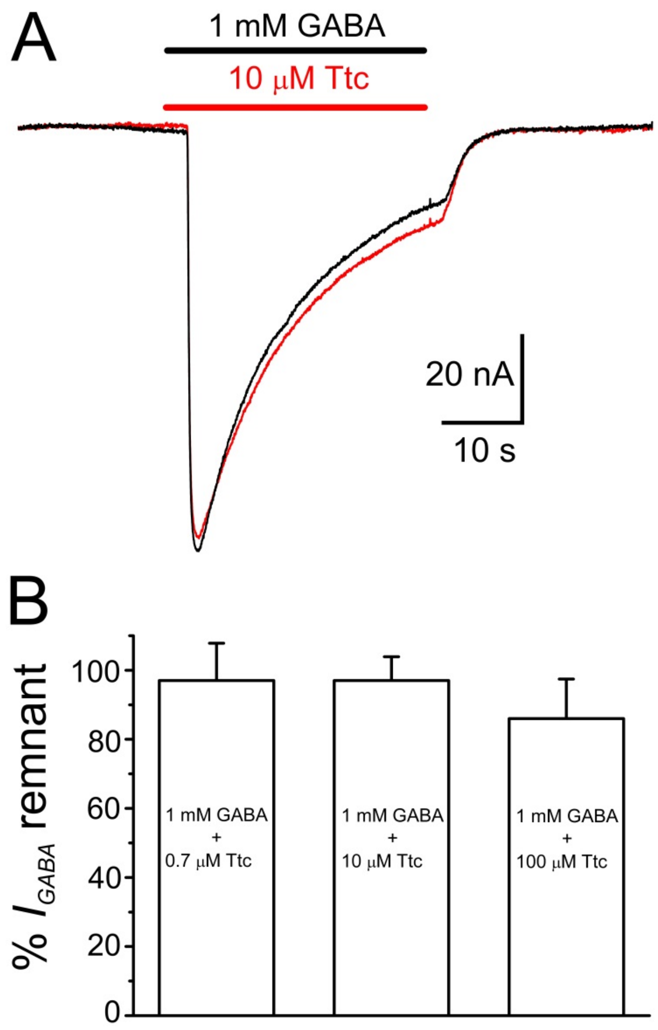

Supplementary Figure S1. *Effect of tetracaine (Ttc) on GABA-elicited currents* (I_{GABA}s). $GABA_A$ receptors ($GABA_A$Rs) from rat brain synaptosomal-enriched membranes were microtransplanted to the *Xenopus* oocyte membrane as previously reported by Alberola-Die *et al*. (Front. Mol. Neurosci. 9:12. doi: 10.3389/fnmol.2016.00012). **(A)** Superimposed I_{GABA}s elicited by superfusing the cell, at -60 mV, with 1 mM GABA either alone (black recording) or together with 10 μM Ttc (red recording). **(B)** Column graph showing the lack of significant effects of Ttc on I_{GABA}, when co-applied with 1 mM GABA, even at Ttc concentrations two orders of magnitude higher than its IC_{50} for muscle-type nAChR blockade. Each column is the average of 6-7 oocytes from 3 frogs.

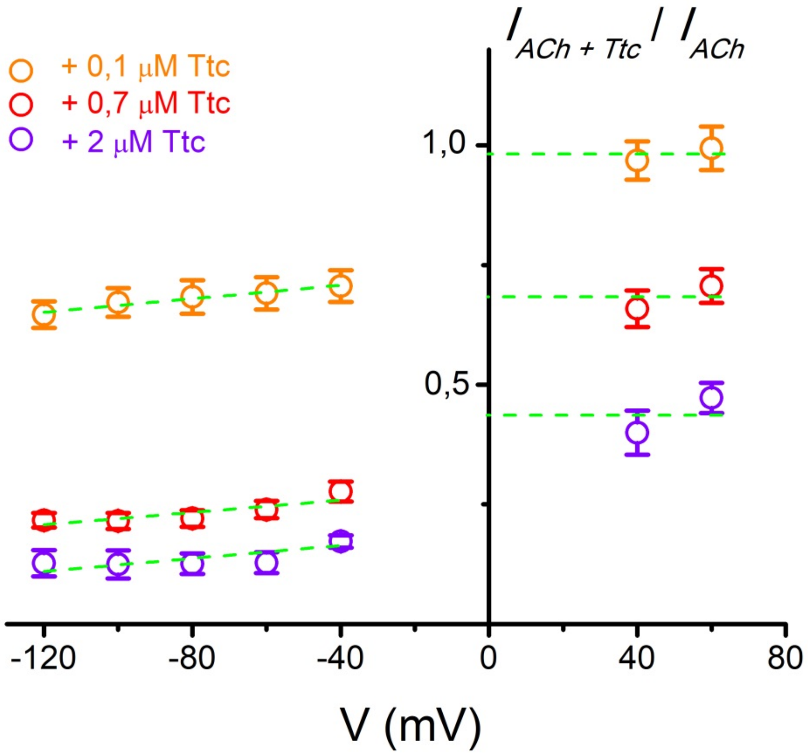

Supplementary Figure S2. *Dose and voltage dependence of the I_{ACh} blockage by Ttc*. Plot of the fraction of the I_{ACh} left by Ttc ($I_{ACh+Ttc}$) at different dosages, normalised to their control I_{ACh}, against the membrane potential, showing the voltage-dependent *nAChR* blockade by Ttc. Notice that, at positive potentials, 0.1 µM Ttc does not elicit I_{ACh} inhibition, but there is a marked nAChR blockade at these potentials when Ttc concentration is raised to 0.7 or 2 µM. Also note that there is a mild voltage dependence at negative potentials at any Ttc concentration tested, suggesting that Ttc binds quite shallow within the channel pore.

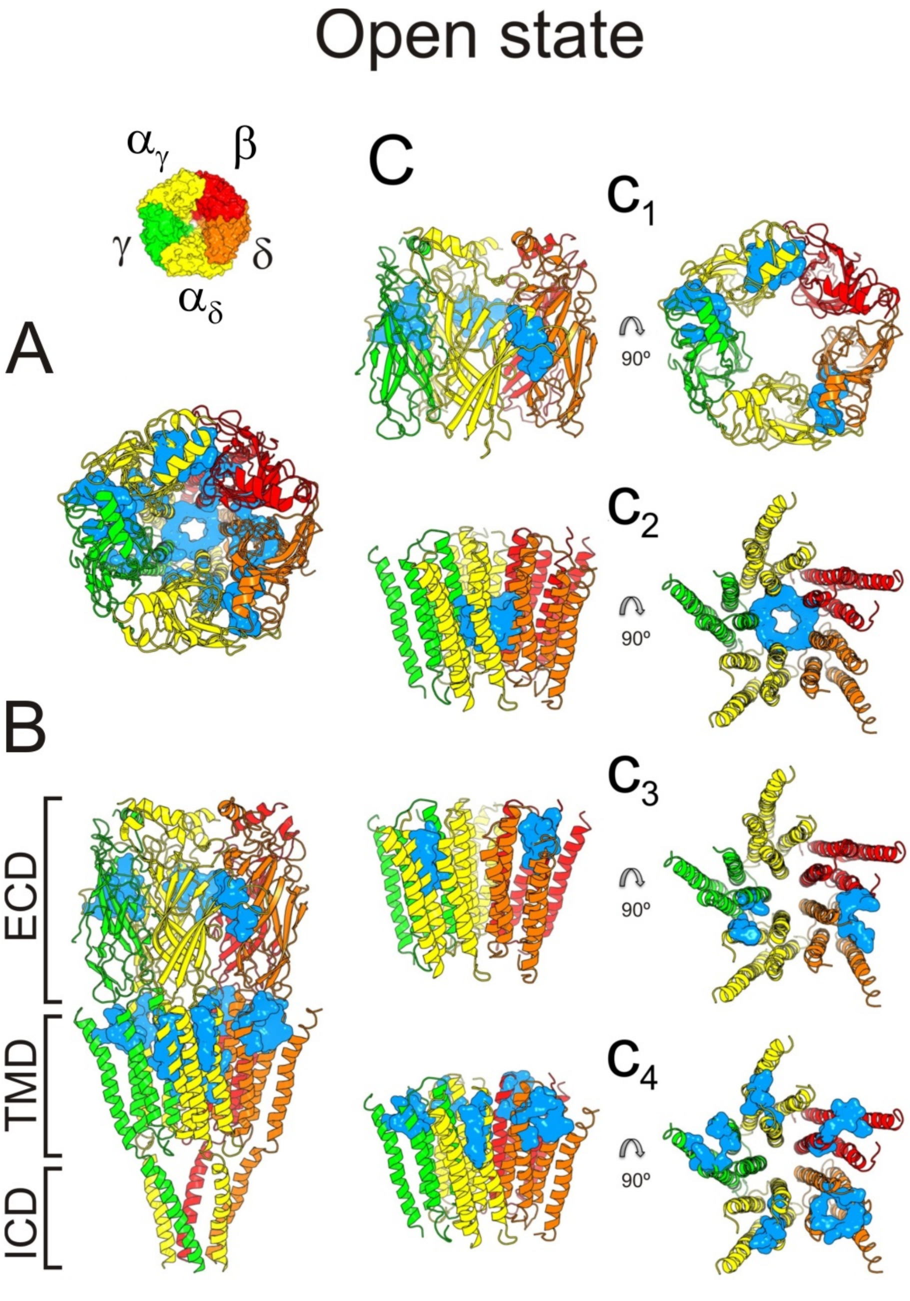

Supplementary Figure S3. *Putative Ttc binding sites at nAChR residues in the open state.* **(A, B)** Ttc binding sites along the entire receptor, highlighted in cyan, as seen from: **(A)** top view of the nAChR, from the synaptic cleft and **(B)** side view, in the membrane plane. Notice that there are Ttc-binding-sites at the extracellular (ECD) and transmembrane (TMD) domains, but not at the intracellular (ICD) one. The inset, in the upper left corner, shows the nAChR subunits with their coding colors. **(C)** More detailed views of Ttc binding sites. Notice that *i)* some Ttc binding sites at the ECD (**c_1**) are in α-γ and α-δ interfaces, i.e., close to the ACh orthosteric site; *ii)* Ttc binding inside the channel (**c_2**) involves all nAChR subunits; and *iii)* most inter-subunit (**c_3**) and intra-subunit (**c_4**) binding sites are placed at crevices in the outer third of the TMD, near the extracellular side.

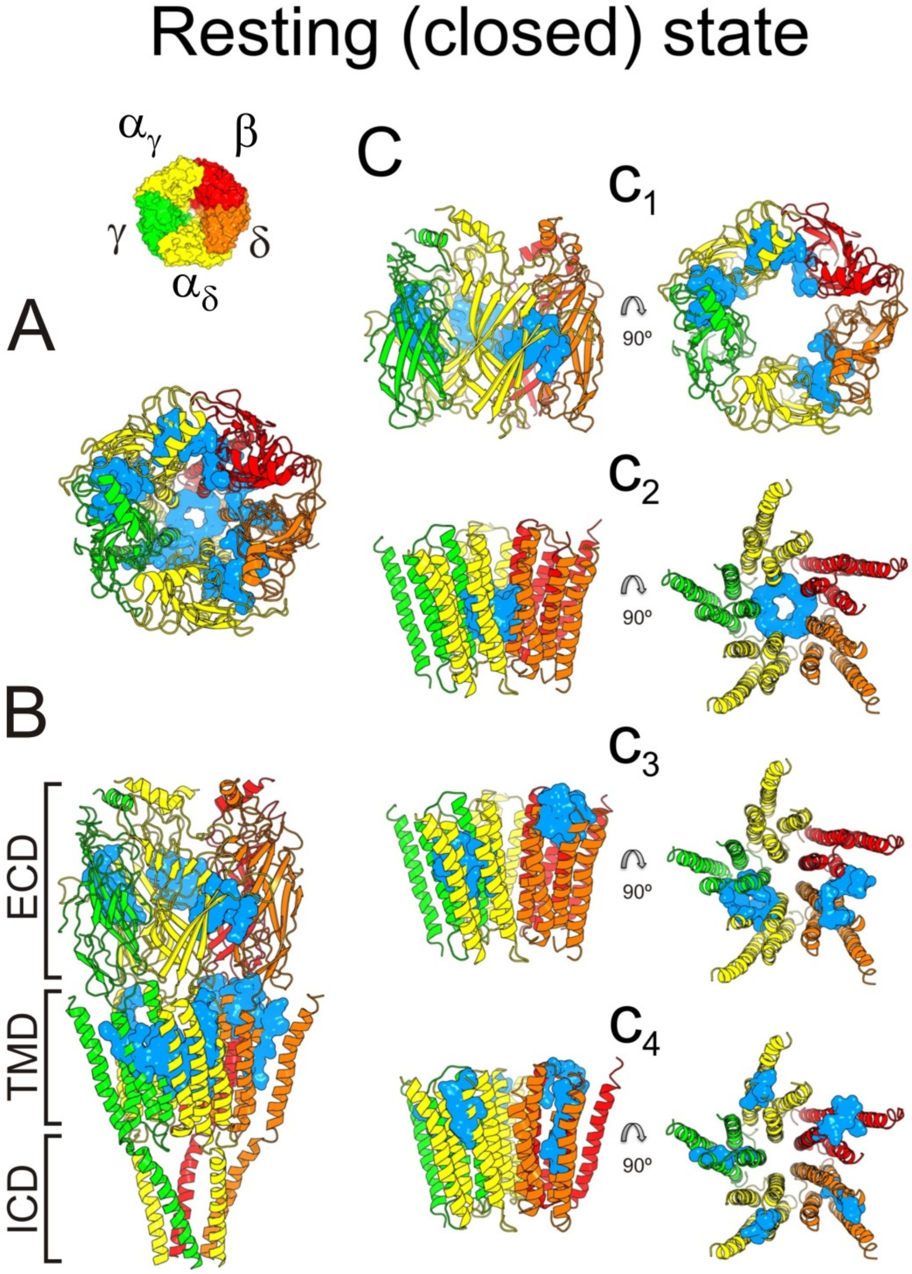

Supplementary Figure S4. *Putative binding sites of Ttc at nAChR residues in the resting state.* Ttc interactions (cyan) with the resting (closed) nAChR observed from top **(A)** and lateral **(B)** views of the whole nAChR. (**C**) More detailed views of Ttc binding sites at ECD and TMD. Ttc interacting sites inside the channel pore (**c_2**) overlapped those of the open conformation. However, there were differences between Ttc-binding residues at the ECD (**c_1**) and at inter- (**c_3**) and intra-subunit (**c_4**) crevices of the TMD of the closed and open nAChRs (see Table 1). Colors are as in Figure S3.

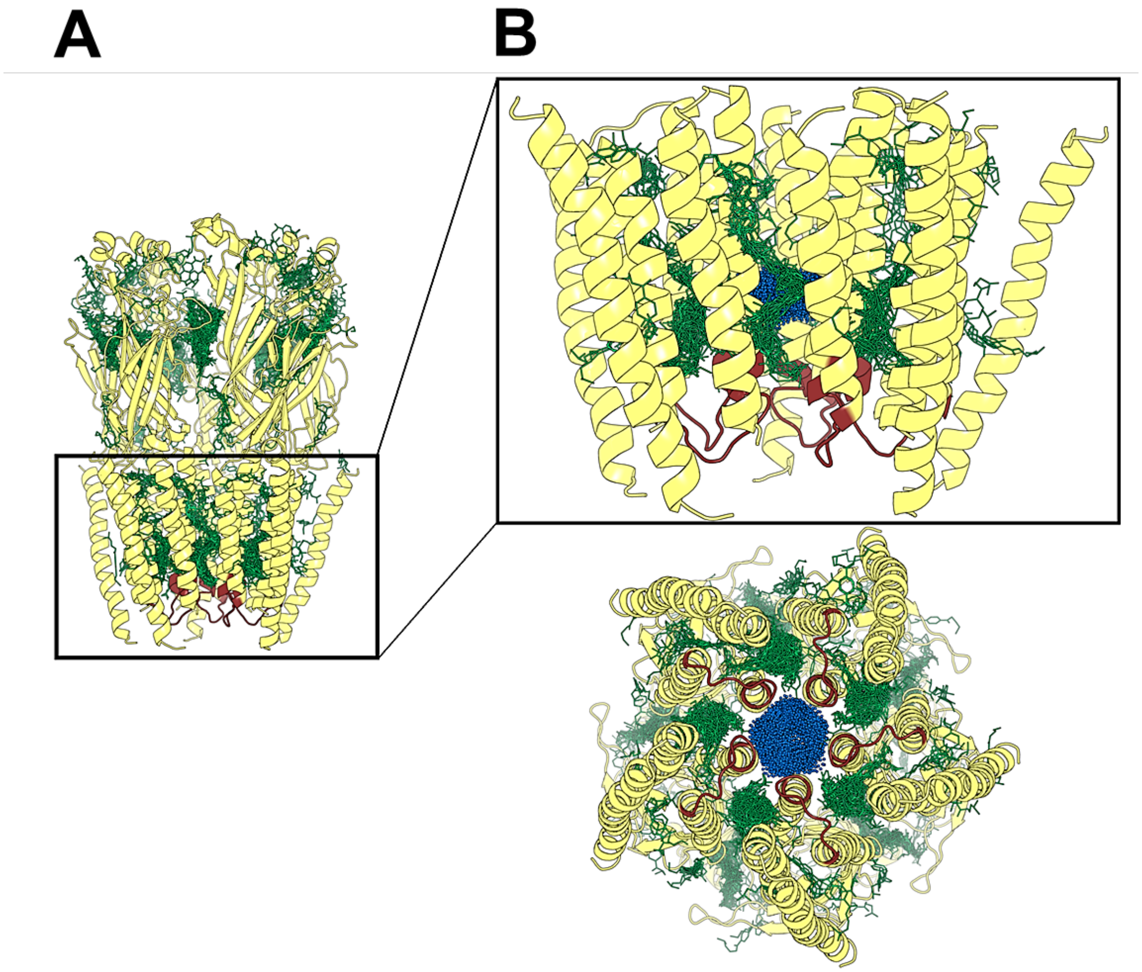

Supplementary Figure S5. *Homomeric alpha-7 receptor in the resting state as a target for Ttc docking.* In the absence of a refined model of the muscular-type nAChR, we has used the refined model of the homomeric α_7-nAChR (Newcombe *et al.*, 2018, doi:10.1124/mol.117.110551) to test the relevance of M1-M2 loop in the Ttc docking. Panel A shows a side view of α_7 (yellow ribbons) interacting with Ttc (green and blue sticks) after 800 docking runs. Loop M1-M2 affected by the refinement is colored in brown. Panel B just shows the TM domain (side and cytoplasmic views). Ttc solutions inside the pore are colored in blue, whereas either inter- or intra-subunit solutions are colored in green. Notice that Ttc does not interact on the M1-M2 loop or nearby residues of the M2 helix.

Receptor state	Binding site location	Interfaces	Mean binding energy (kcal/mol)	K_d (M)	n	Average binding energy (kcal/mol)	K_d (M)
Resting	Site-1 (ECD)	α-γ	5.02 ± 0.48	2.07x10^{-4}	45	4.79 ± 0.54	3.08x10^{-4}
		α-δ	4.50 ± 0.37	4.99x10^{-4}	52		
		α-β	4.84 ± 0.58	2.84x10^{-4}	36		
	Site-2 (TMD near ECD)	α-γ	3.67 ± 0.67	2.05x10^{-3}	12	3.67 ± 0.67	2.05x10^{-3}
	Site-3 (M2)	α-β-δ-α-γ	4.75 ± 0.19	3.31x10^{-4}	66	4.75 ± 0.19	3.31x10^{-4}
	TMD (Intersubunit)	β-δ	5.36 ± 0.54	1.18x10^{-4}	64	5.30 ± 0.63	1.31x10^{-4}
		α-γ	5.24 ± 0.69	1.45x10^{-4}	46		
	TMD (Intrasubunit)	α	4.42 ± 0.45	5.74x10^{-4}	66	4.63 ± 0.53	4.03x10^{-4}
		β	5.06 ± 0.45	1.94x10^{-4}	56		
		δ	4.45 ± 0.30	5.44x10^{-4}	21		
		γ	4.81 ± 0.66	2.95x10^{-4}	42		
Open	Site-1 (ECD)	α-γ	4.79 ± 0.51	3.05x10^{-4}	48	4.53 ± 0.66	4.74x10^{-4}
		α-δ	4.36 ± 0.63	6.31x10^{-4}	42		
		α-β	4.44 ± 0.79	5.52x10^{-4}	17		
	Site-2 (TMD near ECD)	α-γ	3.48 ± 0.36	2.79x10^{-3}	33	3.48 ± 0.36	2.79x10^{-3}
	Site-3 (M2)	α-β-δ-α-γ	3.94 ± 0.51	1.28x10^{-3}	58	3.94 ± 0.51	1.28x10^{-3}
	TMD (Intersubunit)	β-δ	4.26 ± 0.48	7.50x10^{-4}	26	4.82 ± 0.76	2.90x10^{-4}
		α-γ	5.39 ± 0.60	1.12x10^{-4}	45		
	TMD (Intrasubunit)	α	4.11 ± 0.28	9.70x10^{-4}	46	4.28 ± 0.61	7.22x10^{-4}
		β	5.03 ± 0.48	2.04x10^{-4}	59		
		δ	3.96 ± 0.48	1.24x10^{-3}	27		
		γ	4.20 ± 0.28	8.31x10^{-4}	39		

Supplementary Table S1. *Estimated binding energies and dissociation constants (K_d) of Ttc-nAChR interactions in resting- and open-receptor conformations*. The preferential conformations of Ttc bound to single locations are listed together with their binding energies (mean

and standard deviation) and their corresponding K_ds (n is the number of docking solutions averaged). Data from different subunit locations in the same domain were averaged to get a single energy value. Location in the nAChR of Ttc binding sites “1”, “2” and “3” is shown in Table 2 inset (see main text). The remaining Ttc binding sites are displayed in Supplementary Figures S3 and S4.
